# Improving the ameliorative effects of berberine and curcumin combination via dextran-coated bilosomes on non-alcohol fatty liver disease in mice

**DOI:** 10.1186/s12951-021-00979-1

**Published:** 2021-08-04

**Authors:** Yi Chen, Zhaohui Jiang, Jinzhuan Xu, Jiyuan Zhang, Runbin Sun, Jia Zhou, Yuan Lu, Zipeng Gong, Jing Huang, Xiangchun Shen, Qianming Du, Jianqing Peng

**Affiliations:** 1 State Key Laboratory of Functions and Applications of Medicinal Plants, Guizhou Medical University, Guiyang, 550014 China; 2 Key Laboratory of Optimal Utilization of Natural Medicine Resources, School of Pharmaceutical Sciences, Guizhou Medical University, Guiyang, 550025 China; 3 General Clinical Research Center, Nanjing First Hospital, Nanjing Medical University, Nanjing, 210006 China; 4 Department of Clinical Pharmacy, School of Basic Medicine & Clinical Pharmacy, China Pharmaceutical University, Nanjing, 210009 China; 5 Department of Clinical Laboratory, The First People’s Hospital of Guiyang, Guiyang, 550002 China; 6 Nanjing Drum Tower Hospital, the Affiliated Hospital of Nanjing University Medical School, Nanjing, 210008 China

**Keywords:** Berberine, Curcumin, Bilosomes, DEAE-DEX, Oral delivery, NAFLD

## Abstract

**Background:**

The combination of berberine (BER) and curcumin (CUR) has been verified with ameliorative effects on non-alcohol fatty liver disease (NAFLD). However, discrepant bioavailability and biodistribution of BER and CUR remained an obstacle to achieve synergistic effects. Multilayer nanovesicles have great potential for the protection and oral delivery of drug combinations. Therein lies bile salts inserted liposomes, named as bilosomes, that possesses long residence time in the gastrointestinal tract (GIT) and permeability across the small intestine. Diethylaminoethyl dextran (DEAE-DEX) is generally used as an outside layer on the nanovesicles to increase the mucinous stability and promote oral absorption. Herein, we developed a DEAE-DEX-coated bilosome with BER and CUR encapsulated (DEAE-DEX@LSDBC) for the treatment of NAFLD.

**Results:**

DEAE-DEX@LSDBC with 150 nm size exhibited enhanced permeation across mucus and Caco-2 monolayer. In vivo pharmacokinetics study demonstrated that DEAE-DEX@LSDBC profoundly prolonged the circulation time and improved the oral absorption of both BER and CUR. Intriguingly, synchronized biodistribution of BER and CUR and highest biodistribution at liver was achieved by DEAE-DEX@LSDBC, which contributed to the optimal ameliorative effects on NAFLD. It was further verified to be mainly mediated by anti-oxidation and anti-inflammation related pathways

**Conclusion:**

DEAE-DEX coated bilosome displayed promoted oral absorption, prolonged circulation and synchronized biodistribution of BER and CUR, leading to improved ameliorative effects on NAFLD in mice, which provided a promising strategy for oral administration of drug combinations.

**Graphic abstract:**

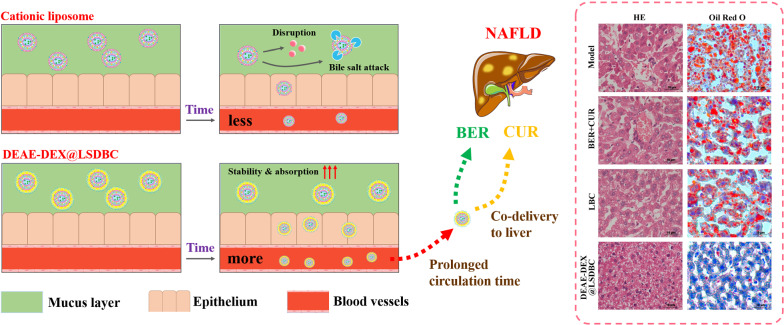

**Supplementary Information:**

The online version contains supplementary material available at 10.1186/s12951-021-00979-1.

## Background

Non-alcohol fatty liver disease (NAFLD) is characterized hepatic steatosis in the absence of heavy alcohol consumption that caused by the overuse of hepatotoxic medication, disorders affecting the liver such as hepatitis C virus infection, Wilson’s disease and starvation [1, 2]. It had affected approximately 25% of the global population and high prevalence was observed in patients with metabolic derangements [3]. One third of NAFLD patients might develop into nonalcoholic steatohepatitis (NASH), which associates with steatosis, hepatocyte swelling and lobular inflammation, resulting in liver fibrosis and cirrhosis, even hepatic failure and hepatocellular carcinoma (HCC) [4]. Given that hepatic failure is irreversible, amelioration of NAFLD on time to prevent the liver fibrosis and reduce the occurrence of hepatitis-related and extrahepatic diseases can effectively diminish the mortality induced by hepatic failure [5]. However, due to the not fully understood pathogenesis, there is a lack of ideal drugs for the treatment of NAFLD. So far, the multiple-hit hypothesis for the pathogenesis of NAFLD has been widely accepted. It holds that the development and progression of NAFLD resulted from the multiple damages, including insulin resistance, adipose tissue dysfunction, intestinal flora disorders, oxidative stress damage, genetic and epigenetic factors [6, 7]. Since the complexity of NAFLD as a metabolic disease, it is necessary and highly effective to apply multitarget therapies on it.

In recent years, many herbal extracts have been proved with great potential for the treatment of NAFLD, including flavonoids, alkaloids, polysaccharides, volatile oils, quinones, terpenes, coumarins, lignans, saponins, cardiac glycosides, phenolic acids, and amino acids [8]. These extracts alleviated lipid infiltration at liver and improved related anthropometric, hemodynamic and biochemical parameters via immunomodulatory, antioxidant, anti-inflammatory, and anti-fibrosis properties with low toxicity [8, 9]. Among them, berberine (BER) and curcumin (CUR) exhibited promising ameliorative therapeutic effects on NAFLD.

BER as an isoquinoline alkaloid extracted from *Coptis chinensis* has been used for diarrhea, inflammatory diseases, and metabolic disorders including obesity and diabetes [10]. Although the mechanisms of BER in the treatment of NAFLD are unclear, a variety of potential targeting pathophysiological processes has been proposed, including increase of hepatic insulin sensitivity [11], reduction of serum cholesterol by stabilizing mRNA of low density lipoprotein receptor (LDLR) [12], enhancement of mitochondrial function to reduce oxidative stress [13] and regulation on adenosine monophosphate activated protein kinase (AMPK) pathway [14]. Similar but not identical, CUR as a natural polyphenol isolated from *Curcuma longa* has diverse effects of anti-oxidation, anti-inflammation, anti-mutagenesis, anti-fibrosis and reduction on the liver steatosis [15, 16]. The combination of BER and CUR exerted superior therapeutic effect on NAFLD in rats in contrast to the single drug or lovastatin (a traditional cholesterol-lowering agent) by regulating lipid metabolism, reducing oxidative stress and ameliorating liver inflammation [17]. However, the combination of BER and CUR remains unsatisfied due to the potential toxicity induced by high dosage, especially BER-induced gastrointestinal tract (GIT) damages at higher than 50 mg/kg in rats via oral administration [18]. The low oral absorption, rapid metabolism and enhanced excretion of BER and CUR are the main reasons for the poor oral bioavailability, which limited the pharmacological actions of the combination [19–21]. Therefore, a co-delivery nanoplatform for BER and CUR combination is highly in need for promoting the ameliorative effects on NAFLD with low oral dosage via improved oral absorption, prolonged circulation and synchronized biodistribution to liver.

In terms of nanoplatforms for oral co-delivery of drugs, liposomes were considered as efficient vehicles for both hydrophilic and hydrophobic via interactions between liposomes and intestinal cells leading to membrane fusion and endocytosis [22, 23]. Although cationic liposome was verified with higher cellular uptake and transportation across intestinal cells integrally in comparison with anionic or neutral liposomes, it failed to maintain the integrity of the majority of liposomes in GIT before absorption resulting in drug leakage [24, 25]. Therefore, liposomal carriers with better stability and longer residence time in the GIT have been researched. Bile salts are generally used to reinforce liposomes against complex GIT environment, which named as bilosomes. The insertion of bile salts could effectively stabilize the lipid bilayer against the destruction mainly from endogenous bile acids, thus prolonged the residence time of bilosomes in the GIT [26]. Meanwhile, the presence of bile salts enhanced absorption of bilosomes across the intestinal membranes via both trans-enterocytic internalization and paracellular permeation [27]. There is good correlation between particles size of liposomal preparations and the permeation ability. A particle size less than 400 nm is preferred regardless of the trans-enterocytic internalization and paracellular permeation [28]. Moreover, coating a cover on the surface of liposomes is a good strategy to further prevent the destruction of liposomes in the GIT, thereby maintaining the smaller size and enhancing the stability. Diethylaminoethyl dextran (DEAE-DEX), a cationic polymer, had been used as the cover on the surface of nanoparticles to improve mucinous stability in the GIT fluids, inhibit opsonization in the circulation and promote favorable uptake by hepatocytes via anionic moiety of the asialoglycoprotein receptor (ASGPR) [29, 30]. Besides, cationic polymer-modified liposomes have been reported to prolong residence time via adhesion to intestinal wall mucus as well [31]. Herein, we speculated that the DEAE-DEX coated bilosomes have great potential to protect the bilosomes in the GIT resulting in promoted oral absorption of drugs.

**Scheme 1:**
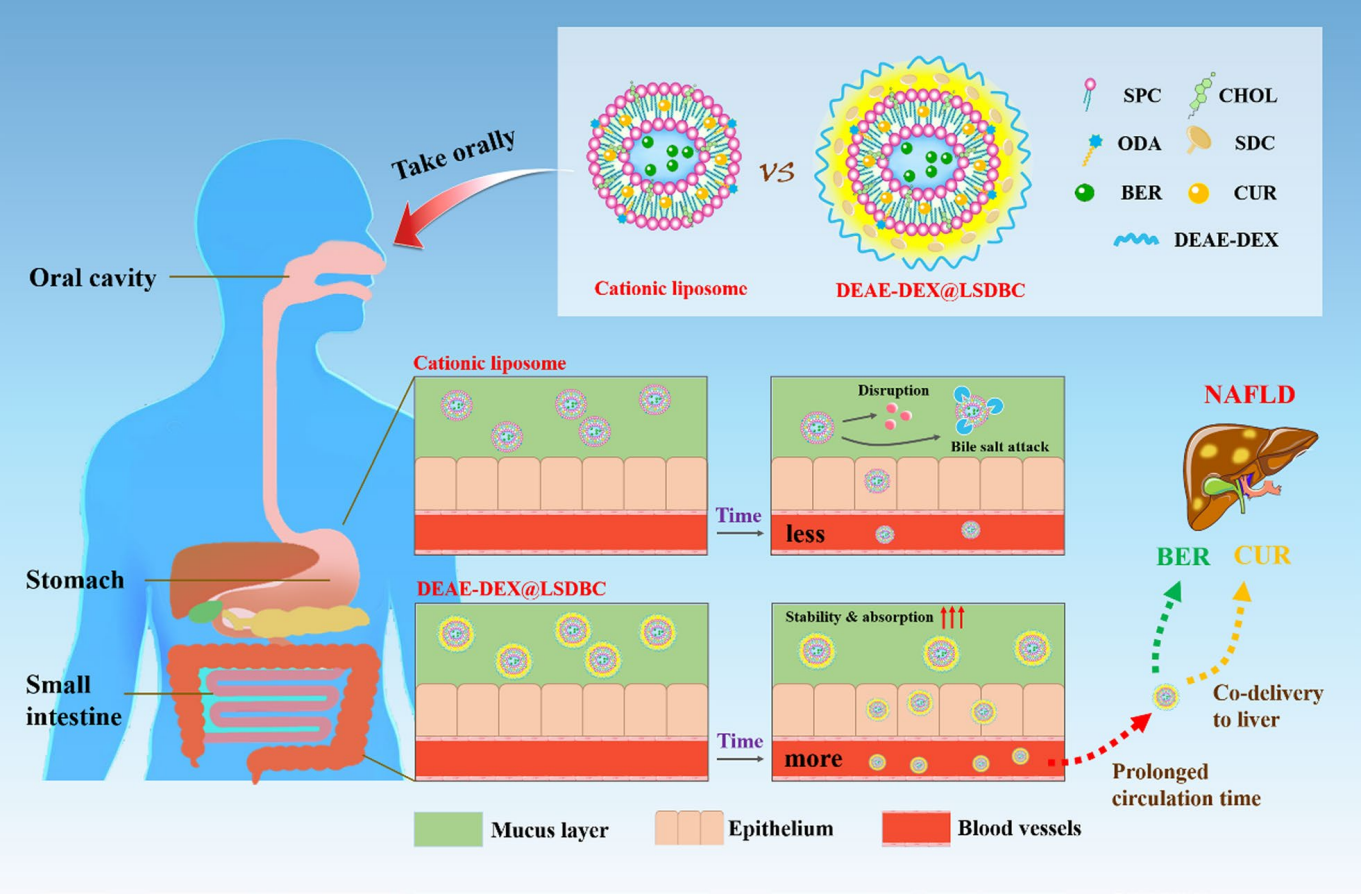
The superiority of DEAE-DEX@LSDBC over cationic liposomes indicated by promoted oral absorption, prolonged circulation and synchronized biodistribution of BER and CUR especially at liver leading to enhanced ameliorative effects on NAFLD

Based on these considerations, we developed DEAE-DEX coated bilosomes that composed of a type of bile salts-sodium deoxycholate (SDC), soybean lecithin (SPC), cholesterol (CHOL), octadecylamine (ODA) with BER and CUR encapsulated (DEAE-DEX@LSDBC) for the treatment of NAFLD via oral delivery (Scheme 1). It realized elevated trans-mucus potential, enhanced absorption, prolonged blood circulation, synchronized biodistribution and improved therapeutic efficacy on NAFLD compared with free BER and CUR combination. Thus, this work proposed an effective multilayer nanovesicles for drug combinations to treat NAFLD via oral administration.

## Results and discussion

### Preparation and optimization of DEAE-DEX@LSDBC

**Fig. 1:**
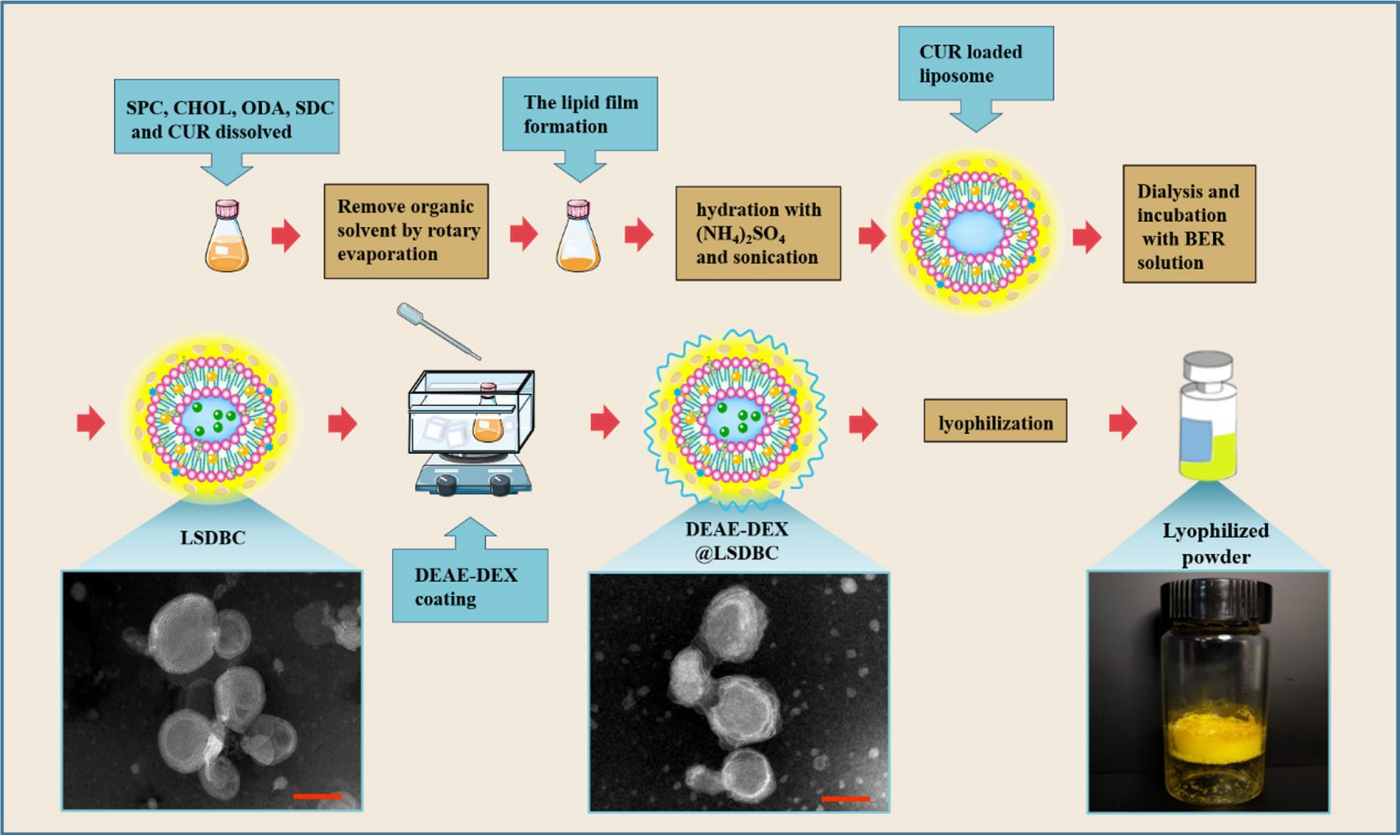
Schematic illustration of the preparation procedure of DEAE-DEX@LSDBC. Typical patterns of preparations are included, such as the TEM images of LSDBC and DEAE-DEX@LSDBC (scale bar 100 nm) and the lyophilized morphology of DEAE-DEX@LSDBC

As a hydrophilic/hydrophobic drug combination, the BER and CUR were encapsulated into bilosomes by a sequential drug loading process to receive LSDBC, in which the common thin-film dispersion was used for CUR followed by a remote loading method for BER. DEAE-DEX was added dropwise into LSDBC under mild stirring to prepare DEAE-DEX@LSDBC. Comparing with LSDBC, a fluffy periphery layer was clearly observed on the outside of DEAE-DEX@LSDBC by TEM. After lyophilization, a yellow spongy solid was obtained (Fig. 1).

Based on the previous developed liposomal preparations for drug combination, several key factors in the formulation and preparation of LSDBC that closely related to the drug loading and characteristics of the bilosomes were optimized. Both the external phase pH and incubation time showed no effect on the encapsulation of CUR, while BER exhibited highest LR and LC as the external phase at pH 7 and incubation for 30 min (Additional file 1: Figure S1). In terms of the SPC:CHOL weight ratio, the encapsulation of CUR was reduced as the ratio of CHOL increased, indicating the competitive relation between CUR and CHOL at the same position in the lipid layer. BER received the highest LC and LR at the SPC:CHOL weight ratio of 10:1, which might be attributed to the optimum flexibility of lipid layer in favor of BER encapsulation in the hydrophilic core. The highest LC of BER was received at a SPC:BER weight ratio of 40:7 reaching the maximum loading capacity, higher concentration of BER reduced both LR and LC on the contrary (Additional file 1: Figure S2).

**Fig. 2:**
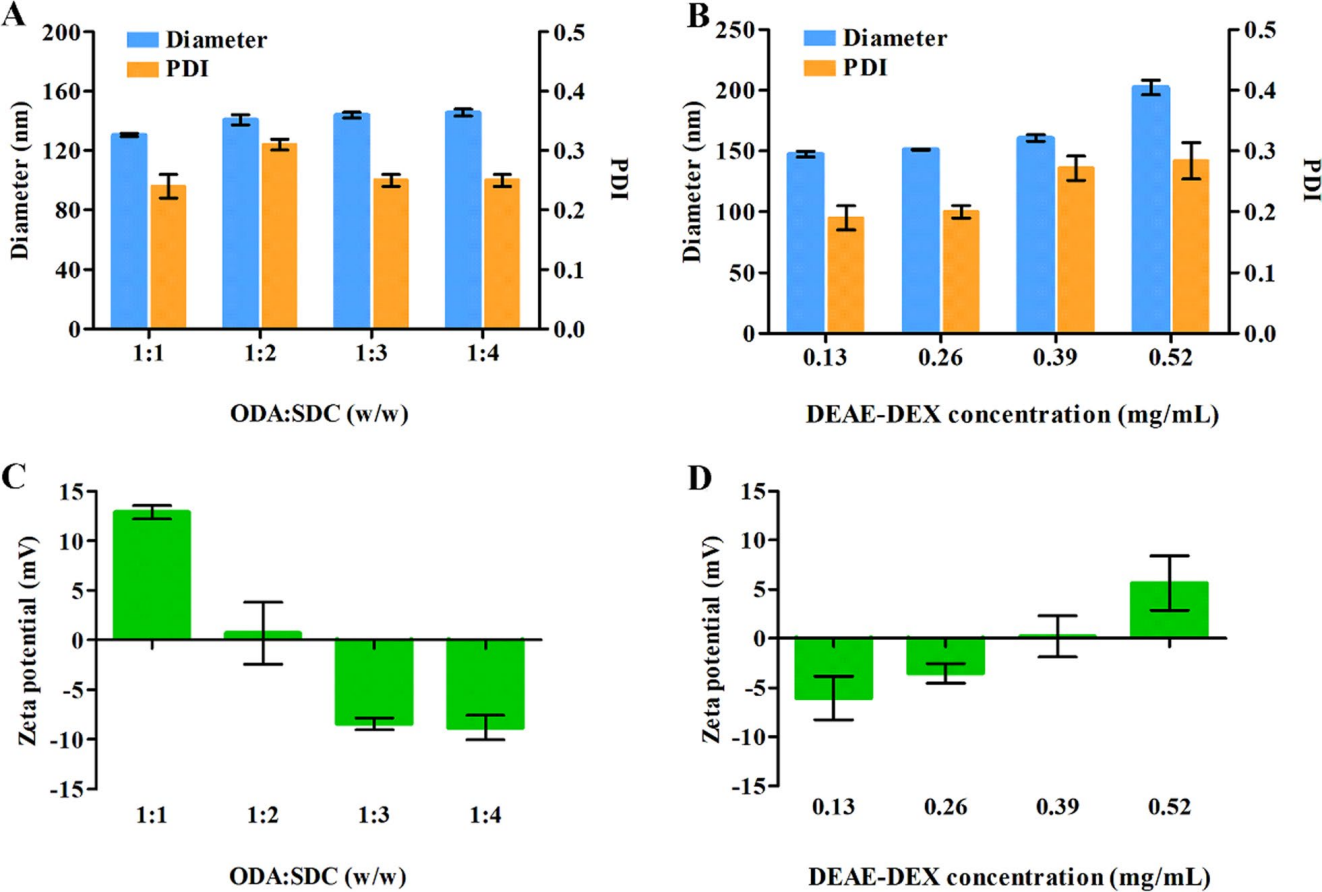
Optimization on the weight ratio of ODA to SDC in terms of **A** diameter, PDI and **C** zeta potential of LSDBC. The concentrations of DEAE-DEX in DEAE-DEX@LSDBC were optimized in terms of **B** diameter, PDI and **D** zeta potential. Each bar represents the mean  ±  SD (*n * =  3)

To steadily absorb DEAE-DEX on the periphery of LSDBC, optimization on the ratio of ODA to SDC is necessary. The decreased molar ratio of ODA:SDC showed little interference on the diameter and PDI of LSDBC, whereas, a charge reversal was detected at 1:3 and no more rise on the negative charge as the ratio decrease (Fig. 2A, C). Thus, the molar ratio of ODA:SDC at 1:3 was used in the preparation of LSDBC to receive negatively charged bilosomes. The content of DEAE-DEX was optimized in terms of diameter, PDI, zeta potential of DEAE-DEX@LSDBC (Fig. 2B, D). Hardly any size variation was observed below 0.39 mg/mL of DEAE-DEX. A charge reversal was detected at 0.39 mg/mL of DEAE-DEX approaching to electrical neutrality. Meanwhile, the highest permeability across mucus layer of DEAE-DEX@LSDBC was detected at the same concentration of DEAE-DEX regardless of the drug (Additional file 1: Figure S3). It confirmed the contribution of hydrophilicity and neutral surface of bilosomes to its mucinous stability. Therefore, the DEAE-DEX at 0.39 mg/mL was adopted to prepare DEAE-DEX@LSDBC.

### Characterization of BER and CUR encapsulated bilosomes

**Fig. 3:**
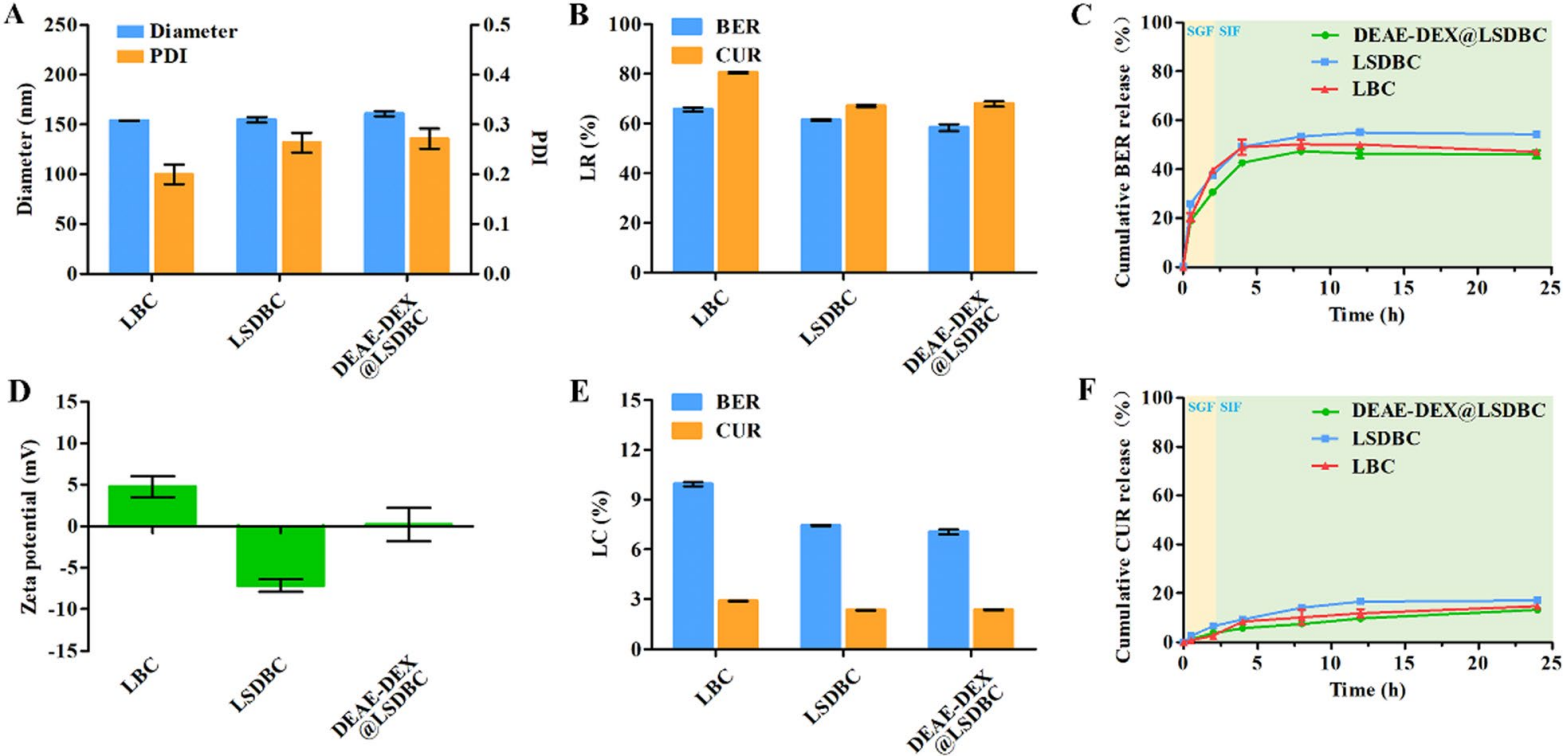
Characterization of the liposomal preparations. **A** Diameter, PDI and **D** Zeta potential of the preparations. **B** LR and **E** LC of the preparations. **C** BER and **F** CUR releases from DEAE-DEX@LSDBC in SFG (0–2 h) and SIF (2–24 h) in vitro. Each bar represents the mean  ±  SD (*n * =  3)

The TEM images showed nearly spherical particles of LSDBC and DEAE-DEX@LSDBC with similar size (Fig. 1). In consistent with the images, the hydrodynamic diameters of LBC, LSDBC and DEAE-DEX@LSDBC measured by dynamic light scattering were approximately 150 nm with a PDI less than 0.3 (Fig. 3A). The charge reversal responding to the SDC insertion and DEAE-DEX coating were detected, respectively (Fig. 3D). Moreover, the UV absorption spectra showed that the characteristic peaks of CUR (425 nm) and BER (352 and 268 nm) were overlapped in drug-encapsulated bilosomes (Additional file 1: Figure S4) indicating the successful encapsulation of BER and CUR. Compared with LBC, the LR and LC of both BER and CUR reduced after the SDC insertion (Fig. 3B, E). It was hypothesized that the insertion of SDC into the lipid bilayer occupied the position of CUR and the negative charge of SDC impeded the encapsulation of BER into internal aqueous phase of bilosomes. Whereas, no more reduction on the LC and LR was detected after DEAE-DEX coating. The final weight ratio of BER and CUR was about 3–1 in all liposomal preparations.

In vitro drug release study was carried out in the SGF and SIF medium under sink condition for both BER and CUR (Fig. 3C, F; Additional file 1: Figure S8). BER showed burst release in the SGF (pH 1.2) medium at initial 1 h and the release rate slowdown in the SIF (pH 6.8) medium reaching 50% cumulative release at 24 h. In comparison with BER, CUR showed lower drug release rate and less than 20% cumulative release at 24 h. It was speculated that the low pH of SIF reduced the gel-like precipitate formed between BER and SO_4_^2−^ in the inner aqueous phase of bilosomes and promoted the release of BER. While, CUR interacted with lipid bilayer via mainly hydrophobic interactions was hardly affected by the media. Noteworthy, DEAE-DEX@LSDBC exhibited slight retardation on the drug release compared with LSDBC indicating the weak interactions between DEAE-DEX and LSDBC, which guaranteed the shell separation of DEAE-DEX to promote the interactions between LSDBC and intestine.

### Permeation across mucus layer and Caco-2 cell monolayer

**Fig. 4:**
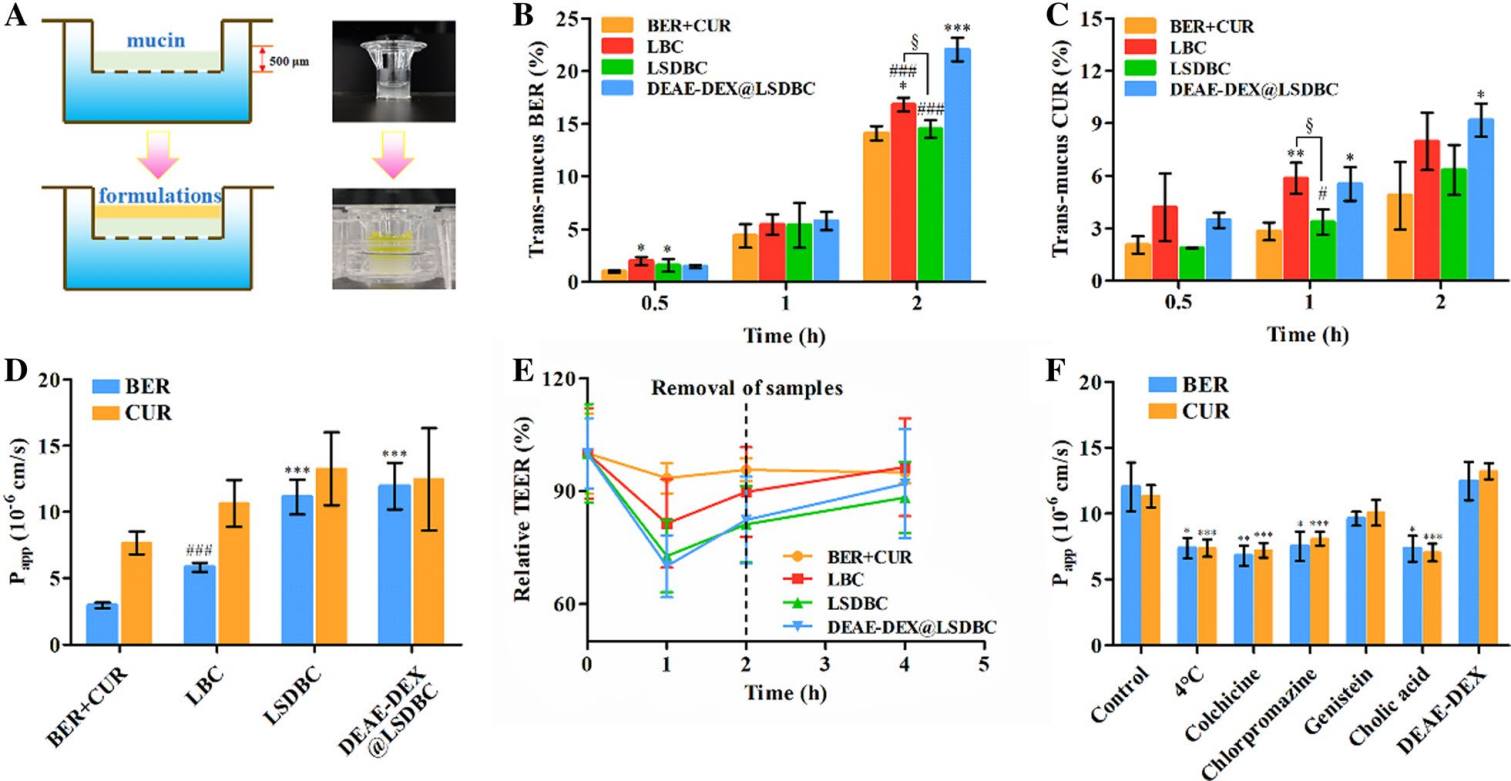
In vitro permeability across mucus layer and Caco-2 cell monolayer. **A** Schematic illustration of the transwell experiment. Mucin (10 mg/mL) was added to the upper chambers (thickness of 500 μm), and the lower cambers were filled with HBSS. Transportation of **B** BER and **C** CUR across mucus layer in different preparations. Each bar represents the mean  ±  SD (*n * =  3). ^*^p  <  0.05, ^**^p  <  0.05 and ^***^p  <  0.001 vs BER  +  CUR; ^#^*p * <  0.05 and ^###^*p * <  0.001 vs DEAE-DEX@LSDBC; ^§^*p*  <  0.05 LBC vs LSDBC*.* **D** P_app_ values of various preparation groups in Caco-2 cell monolayers. Each bar represents the mean  ±  SD (*n * =  3). ^***^p  <  0.001 vs BER  +  CUR; ^###^*p * <  0.001 vs DEAE-DEX@LSDBC*.* **E** Relative TEER changes of the Caco-2 cell monolayers in different groups. After 2 h incubation, the preparations were removed from the Caco-2 cell monolayers. Each bar represents the mean  ±  SD (*n * =  3). **F** P_app_ values of the DEAE-DEX@LSDBC in Caco-2 cell monolayers under different inhibitory conditions. Each bar represents the mean  ±  SD (*n * =  3). ^*^p  <  0.05, ^**^p  <  0.01 and ^***^p  <  0.001 vs Control

After oral administration, the intestinal mucus and enterocytes limited the permeation and transportation of drugs into the systemic circulation. To evaluate the adsorption promotion effects of liposomal preparations on the drug combination, in vitro mucus layer (Fig. 4A–C) and Caco-2 cell monolayer (Fig. 4D–F) was established to mimic the intestinal absorption barriers. At 0.5, 1 and 2 h, the trans-mucus content of BER and CUR were detected. The permeability of BER was obviously promoted with time and DEAE-DEX@LSDBC showed optimal permeability (Fig. 4B). However, the ratio of trans-mucus of CUR exhibited indistinctive time-dependent manner in all the groups and weaker advantages of DEAE-DEX@LSDBC on permeation enhancement (Fig. 4C). We speculated that the neutral surface of DEAE-DEX@LSDBC improved the mucinous stability leading to increased permeability of drugs across mucus layer, especially for BER encapsulated in the inner aqueous phase. The inferior promotion on the trans-mucus percentage of CUR might attribute to the weak protection effects of DEAE-DEX on the lipid bilayer where CUR was inserted.

To evaluate the permeation across enterocytes, the P_app_ values of BER and CUR across Caco-2 cell monolayer were calculated. Above all, all the preparations were verified with less than 40% inhibition on Caco-2 cells for 24 h at the concentration of BER 90 μg/ml and CUR 30 μg/ml (Additional file 1: Figure S5), which was considered with low cytotoxicity for only 2 h incubation in the following experiment. The P_app_ value of BER in LSDBC and DEAE-DEX@LSDBC was approximately four-fold of that of BER  +  CUR, while the P_app_ value of CUR was only 1.7-fold increased. Importantly, the obvious difference between the permeability of BER and CUR was coordinated by LSDBC and DEAE-DEX@LSDBC (Fig. 4D) which provided solid foundation for BER and CUR to maintain the initial ratio of drugs after absorption.

The TEER study was generally used to predict the contribution of paracellular permeation in the oral absorption. As shown in Fig. 4E, all the liposomal preparations on the apical side of monolayers induced immediate reduction in TEER in contrast with no reduction at all for BER  +  CUR. The reduction in TEER was more significant for bilosomes, indicating prominent enhancement of bilosomes on paracellular permeability, which might contribute to the effect of SDC on the tight junctions. Moreover, the gradually increased TEER after removal of preparations indicated recovery of the tight junctions. Thus, it is suggested that bilosomes could transiently and reversibly open the tight junctions in Caco-2 cell monolayers, which is the main mechanism for paracellular permeation. Furthermore, the effect of trans-enterocytic internalization on the permeation promotion of DEAE-DEX@LSDBC was investigated on the Caco-2 cell monolayer (Fig. 4F). Low temperature, colchicine, chlorpromazine and cholic acid significantly suppressed the P_app_ value of both BER and CUR, indicating the trans-enterocytic internalization of DEAE-DEX@LSDBC mainly via micropinocytosis, clathrin and bile acid transporter-mediated transcellular pathways in an energy-dependent process. In short, the promotion of both paracellular permeation and trans-enterocytic internalization contributed to enhance permeation across Caco-2 cell monolayer by bilosomes.

### Pharmacokinetics study on BER and CUR

**Fig. 5:**
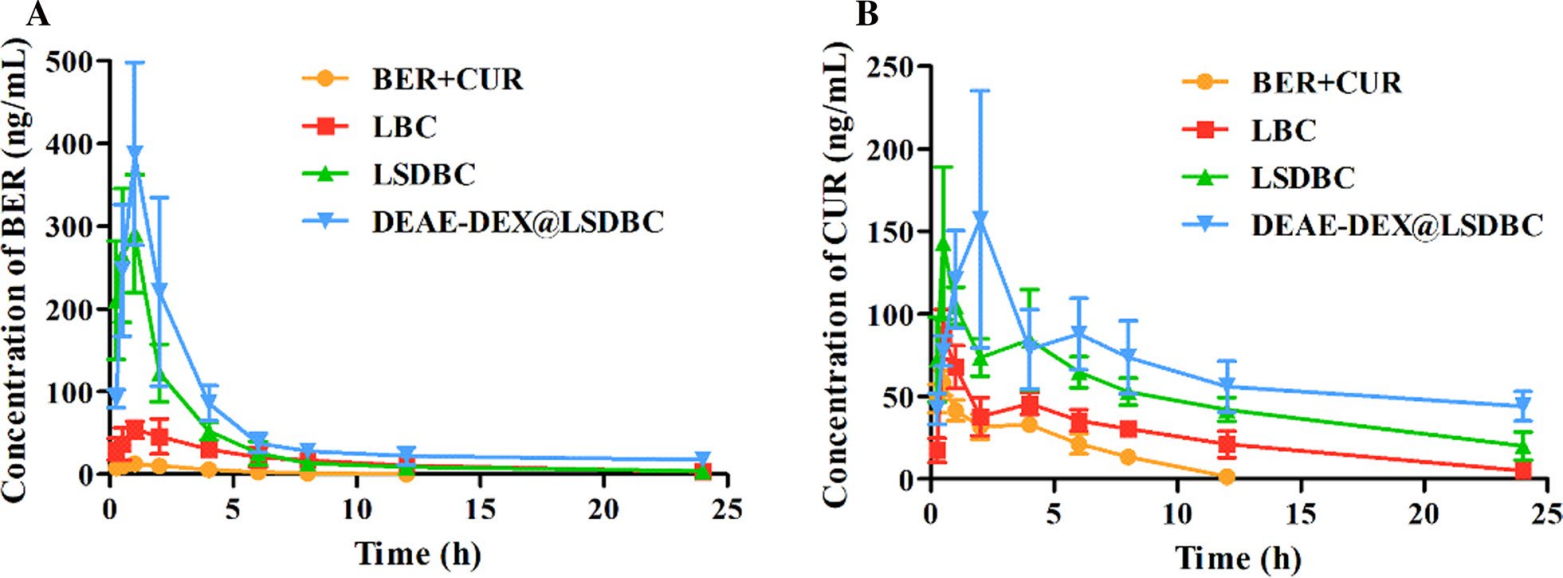
Plasma concentration–time profiles of **A** BER and **B** CUR following the oral administration of various preparations at a dose of 45 mg/kg BER and 15 mg/kg CUR in the male Kunming mice. Each bar represents the mean  ±  SD (*n * =  5)

Oral absorption promotion of BER and CUR with the help of liposomal preparations were verified by the altered plasma concentration-time curves of drugs in the male Kunming mice (Fig. 5). Above all, DEAE-DEX@LSDBC exhibited the best absorption promotion effect on both BER and CUR. LSDBC and DEAE-DEX@LSDBC obviously improved the maximum plasma concentration (C_max_) and area under the curve (AUC) of BER or CUR compared with BER  +  CUR and LBC groups. As a hydrophilic drug, free BER showed extremely flat plasma concentration-time curve. We speculated that the instability of the LBC in GIT fluids might induce disruption on the structure of carrier and leakage of drugs, especially BER loaded in the internal aqueous phase. On the other hand, given that free CUR possessed superior oral bioavailability than free BER, the difference on plasma concentration-time curves narrowed between BER  +  CUR and LBC groups.

Moreover, the major pharmacokinetics parameters of BER and CUR were acquired by the compartmental and non-compartmental model fitting of plasma concentration-curve profiles (Additional file 1: Tables S1; S2). The half-life (T_1/2_) and mean retention time (MRT) of both BER and CUR manifested an excellently prolonged circulation time by the liposomal preparations. DEAE-DEX@LSDBC improved the AUC_0–∞_ of BER and CUR for more than 10 times of the BER  +  CUR. The oral bioavailability of BER was 34-fold increased by DEAE-DEX@LSDBC, while only 13-fold increase was observed in CUR compared with BER  +  CUR. It was reasonable to attribute the superior absorption and longer circulation time of BER to the drug loading position in the inner water phase of bilosomes. The optimal oral absorption promotion and the longest systemic circulation for both BER and CUR was realized by DEAE-DEX@LSDBC. We speculated that the promoted stability of DEAE-DEX@LSDBC in GIT fluids and enhanced permeation across mucus layer and enterocytes contributed to the remarkably promoted oral absorption of BER and CUR.

### In vivo biodistribution of BER and CUR

**Fig. 6:**
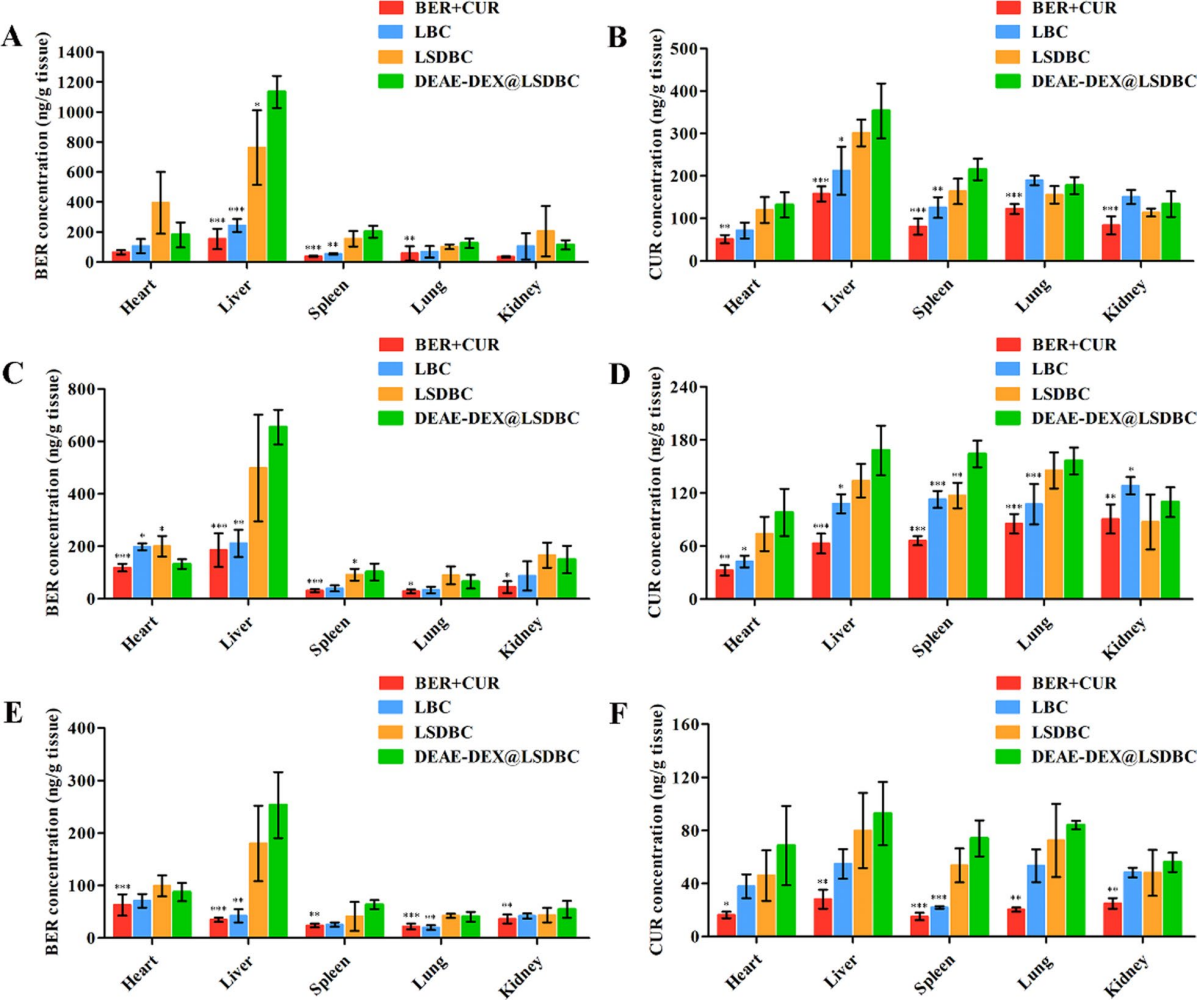
Biodistribution of BER and CUR to each organ after orally administration of BER  +  CUR, LBC, LSDBC and DEAE-DEX@LSDBC at a BER dose of 45 mg/kg for **A** 1 h, **C** 6 h and **E** 24 h and at a CUR dose of 15 mg/kg for **B** 1 h, **D** 6 h and **F** 24 h. Each bar represents the mean  ±  SD (*n * =  3). ^*^p  <  0.05, ^**^p  <  0.01 and ^***^p  <  0.001 vs DEAE-DEX@LSDBC

The biodistribution of BER and CUR from liposomal preparations were further investigated in the male Kunming mice. After oral administration, the major organs were harvested at 1, 6 and 24 h for the drug concentration detection. The concentration of both drugs reached peak at 1 h in each organ and favored the accumulation at liver (Fig. 6A, B). Both drugs showed reduced accumulation in each organ over time in all groups (Fig. 6C–F). It is noteworthy that DEAE-DEX@LSDBC and LSDBC obviously increased the accumulation of BER and CUR at liver compared with BER  +  CUR and LBC, especially for BER. Intriguingly, the content of BER is 3.2-fold of CUR at liver in DEAE-DEX@LSDBC group at 1 h, which is a little higher than the initial ratio of encapsulated BER and CUR that might be attributed to the better promotion effects on BER loading in the inner phase of bilosomes. DEAE-DEX@LSDBC exhibited the optimal liver targeting capability in terms of the BER and CUR, although no significant difference was observed between DEAE-DEX@LSDBC and LSDBC after 6 h. In contrast, the content of BER and CUR were comparative in liver at 1 h between BER  +  CUR and LBC groups. It is suggested that the DEAE-DEX@LSDBC effectively maintained integrity during oral delivery and achieved highest biodistribution of BER and CUR at liver via enhancing the oral absorption and promoting liver accumulation.

Given that synchronized biodistribution of BER and CUR at targeting organs is of great importance to realize the synergistic effects of the combination, the tissue to plasma (T/P) ratio of drug was further calculated to evaluate the synchronism biodistribution in vivo (Additional file 1: Figure S6). All the liposomal preparations showed comparative T/P ratios of BER and CUR, especially at 1 h, which would benefit the amelioration effects of drug combination on NAFLD. However, the difference between the T/P ratio of BER and CUR in the BER  +  CUR group was magnified over time. It is reasonable to suggest that the different in vivo destiny between BER and CUR was efficiently coordinated by the liposomal carriers regardless of the SDC insertion and DEAE-DEX coating.

### Ameliorative effects of the preparations on NAFLD in mice

Above all, the anti-lipid accumulation effects of free BER, CUR, BER  +  CUR and bilosomes of the combination were detected on the free fatty acids (FFA) cultured LO2 cell model. All the preparations at an equivalent concentration of BER 3 μg/ml and CUR 1 μg/ml that was used in the anti-lipid accumulation study exhibited less than 10% inhibition effect on LO2 cells (Additional file 1: Figure S5). The Oil-red-O staining was used to visualize anti-lipid accumulation effects (Additional file 1: Figure S7). It was shown that BER  +  CUR, LBC, LSDBC and DEAE-DEX@LSDBC treatments caused remarkable reduction on the fat accumulation in the cytosol of LO2 cells, while BER and CUR alone possessed inferior anti-lipid accumulation effect in comparison with the BER and CUR combination.

**Fig. 7:**
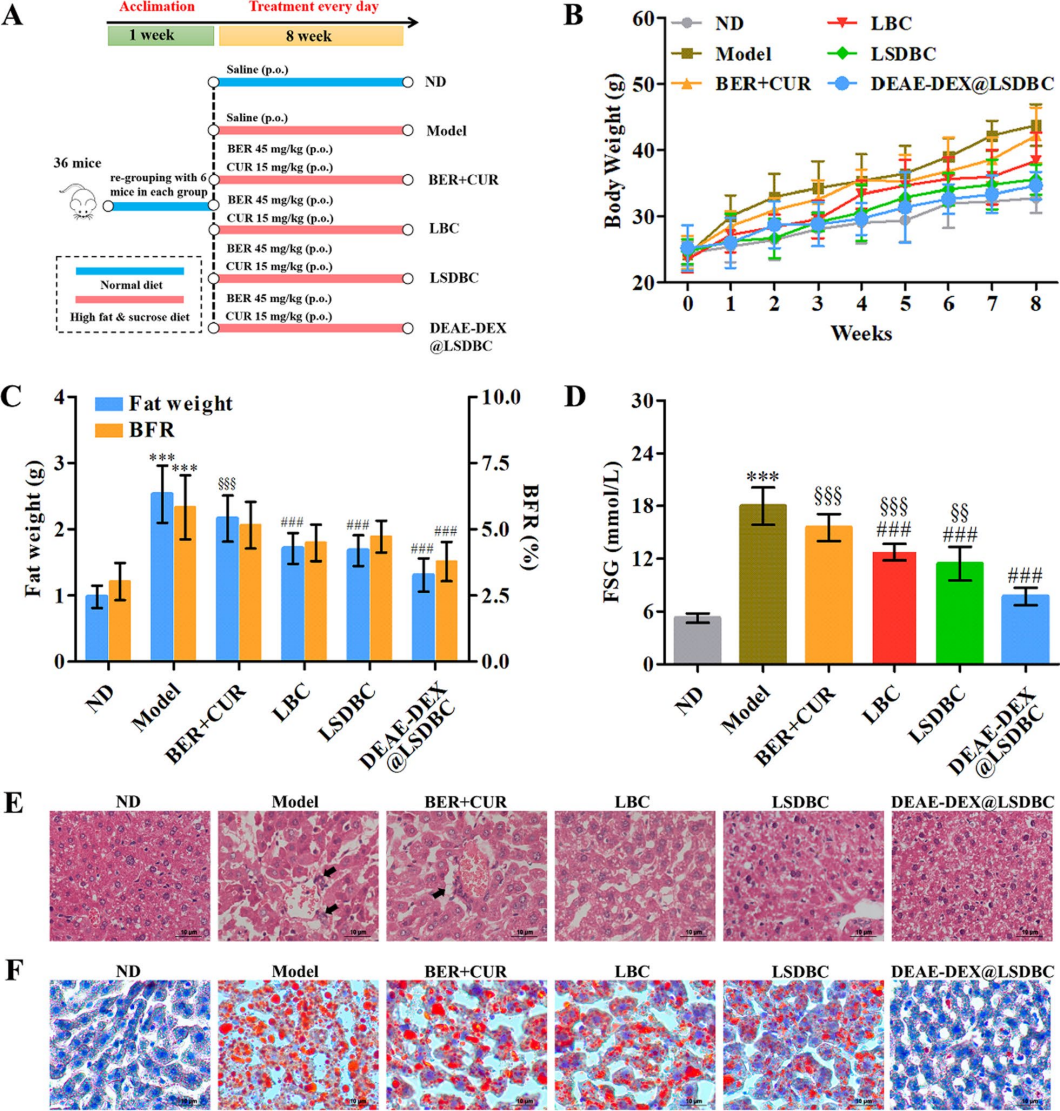
Evaluation of amelioration effects on NAFLD in mice after treatments. **A** Schematic diagram of the animal grouping and treatment. **B** Variation on body weight of mice during the treatments. **C** Detection of fat weight and BFR and **D** FSG at the end of experiments. **E** The H&E staining (black arrow indicates inflammatory foci). **F** Oil-Red-O staining with hematoxylin counterstain. Each bar represents the mean  ±  SD (*n * =  6). ^***^p  <  0.001 Model vs ND; ^###^*p * <  0.001 vs Model; ^§§^*p * <  0.01 and ^§§§^*p * <  0.001 vs DEAE-DEX@LSDBC

NAFLD mice model was established with high fat & sucrose diet and preventively treated by BER and CUR combination following the regimen (Fig. 7A). We monitored the body weight every week and measured the fat weight at the end of the feeding study (Fig. 7B, C). Since the obesity is closely associated with NAFLD, gradually increased body weight of Model group might be one of the indicators for successful model establishment. It was further confirmed by the fat weight detected at the end of experiment. LSDBC and DEAE-DEX@LSDBC showed more flat body weight-time curve, which was in consistent with the lower fat weight. The optimal protective effect of DEAE-DEX@LSDBC on inhibiting fat accumulation was clearly verified by the BFR (Fig. 7C). In addition to fat accumulation at liver, the insulin resistance was recognized as one of the first hit of NAFLD genesis. Therefore, the FSG level after treatments was determined to evaluate the effect of BER and CUR combination on the glucose metabolism. All the treatment groups showed decreased FSG at the end of study although it was still higher than normal group. DEAE-DEX@LSDBC and LSDBC exerted better hypoglycemic effect in contrast to BER  +  CUR and LBC groups (Fig. 7D).

**Fig. 8:**
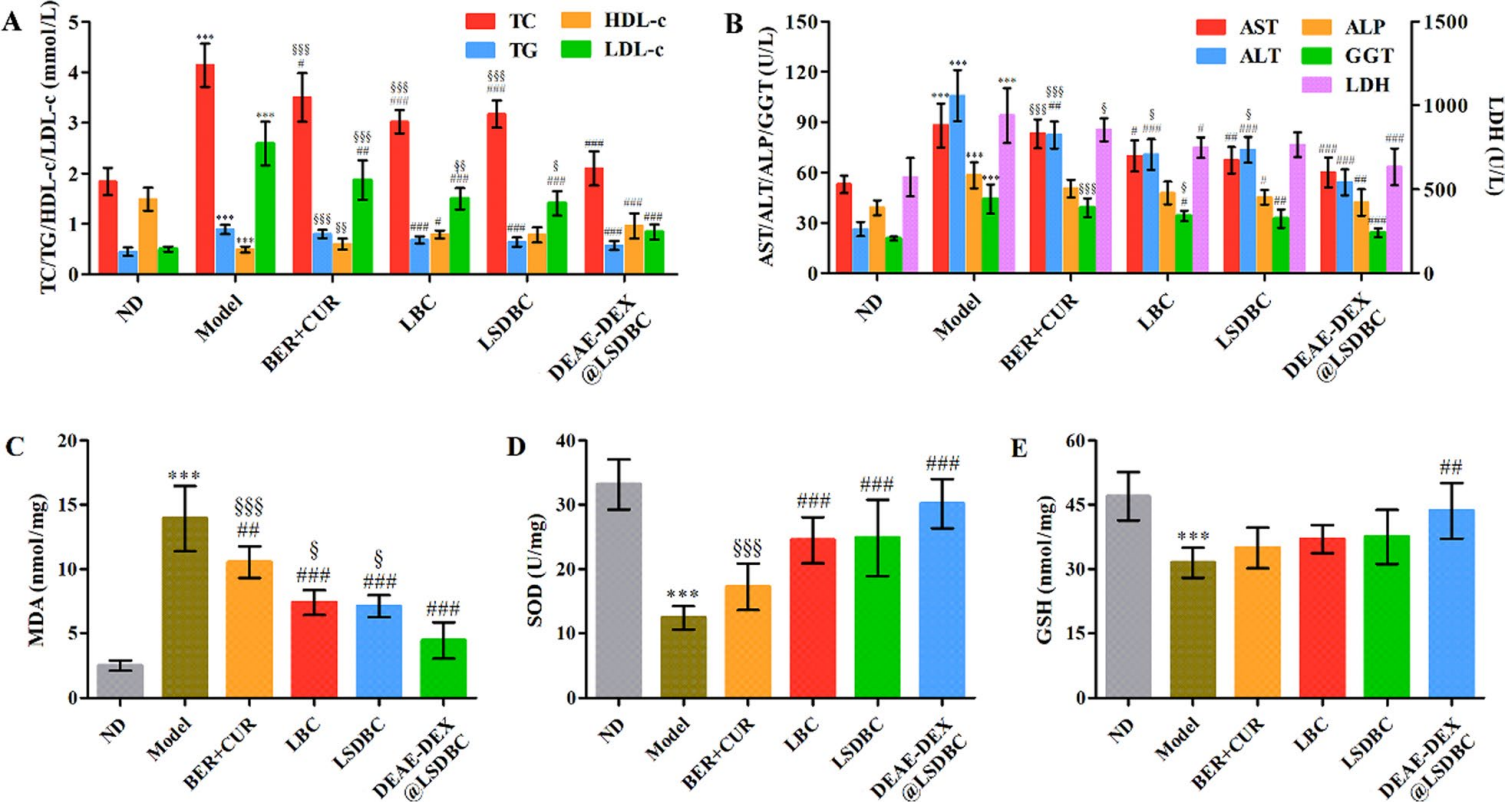
The biochemical results of different treatment groups. **A** The serum lipids indicators including TC, TG, HDL-c and LDL-c. **B** The liver function parameters including AST, ALT, ALP, GGT and LDH. Antioxidant indicators including **C** MDA, **D** SOD and **E** GSH. Each bar represents the mean  ±  SD (*n * = 6 ). ^***^p  <  0.001 Model vs ND; ^#^*p * <  0.05, ^##^*p * <  0.01 and ^###^*p * <  0.001 vs Model; ^§^*p * <  0.05, ^§§^*p * <  0.01, and ^§§§^*p * <  0.001 vs DEAE-DEX@LSDBC

Histopathology analysis on the liver has been used as the golden standard for identification of fatty liver. HE (Fig. 7E) and Oil Red O staining (Fig. 7F) verified the widespread lipid vacuoles and inflammatory foci on the liver of mice in the Model group. At the oral dosage of BER 45 mg/kg and CUR 15 mg/kg, DEAE-DEX@LSDBC and LSDBC exhibited significantly reduced lipid vacuoles and lesion of inflammation compared with model and BER  +  CUR group. LBC showed slight reduced lipid vacuoles, indicating inferior ameliorative effect on the fat accumulation than the bilosomes. DEAE-DEX@LSDBC treatment received the most conspicuous protective effect via preventing lipid droplet accumulation and ameliorating inflammatory injury. Moreover, the serum lipids including TC, TG, HDL-c and LDL-c were measured (Fig. 8A). The drug combination treatments significantly decreased the level of TC, TG, LDL-c and slightly improved the HDL-c level, indicating that BER and CUR combination effectively ameliorated the serum lipid accumulation, especially for DEAE-DEX@LSDBC group. Meanwhile, the degree of liver injury was generally evaluated by the level of AST, ALT, ALP, GGT and LDH (Fig. 8B). The free drug combination and liposomal preparations treatments reduced the level of all the indicators to different degrees. In the DEAE-DEX@LSDBC group, all the indicators approached to the normal mice suggesting the more effective protective effects on liver. Besides, oxidative stress as one of the major promoting roles for NAFLD genesis in mice was evaluated by the serum MDA, SOD and GSH levels (Fig. 8C–E). Compared with the Model group, reduced MDA or improved SOD and GSH at liver in the treatment groups, especially in DEAE-DEX@LSDBC, indicating the highly effective ameliorative effects on oxidative stress in mice induced by the high fat and sucrose diet. Collectively, BER and CUR combination in liposomal preparations have been proved to enhance the ameliorative effects on NAFLD in mice, especially in DEAE-DEX@LSDBC.

### Protein levels related to oxidative stress and inflammation in liver

**Fig. 9:**
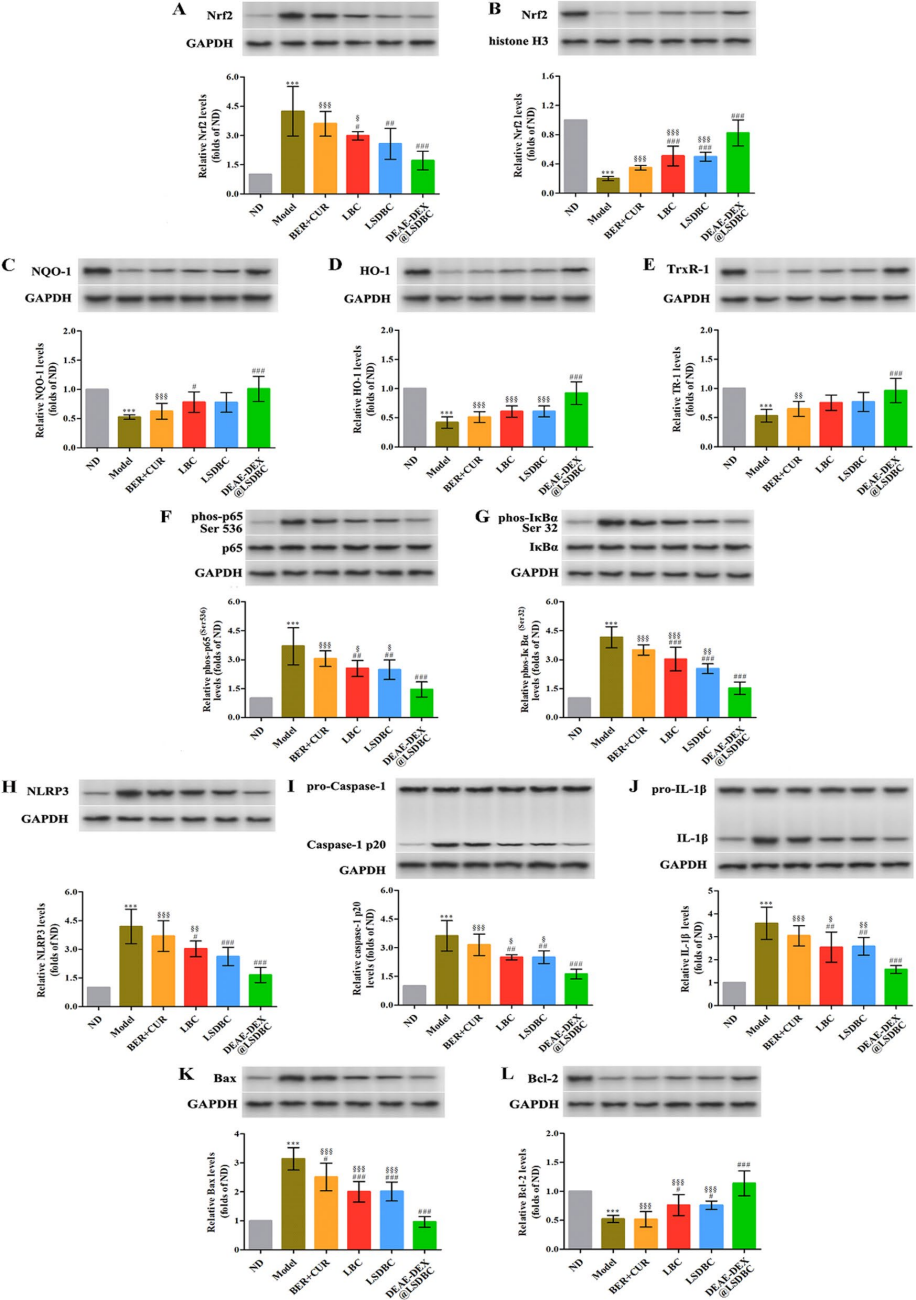
DEAE-DEX@LSDBC activates the **A**–**E** Nrf2 signaling, inhibits the **F**–**J** NLRP3 inflammasome activation and **K**, **L** apoptosis. The indicated protein levels from different groups were detected by western blot and statistical graphs show the relative expression levels of proteins. Each bar represents the mean  ±  SD (*n * =  6). ^***^p  <  0.001 Model vs ND; ^#^*p * <  0.05, ^##^*p * <  0.01 and ^###^*p * <  0.001 vs Model; ^§^*p*  <  0.05, ^§§^*p * <  0.01 and ^§§§^*p * <  0.001 vs DEAE-DEX@LSDBC

To figure out the mechanisms involved in the ameliorative effects of BER and CUR combination treatments on NAFLD in mice, the level of typical proteins related to oxidative stress and inflammation were measured, since the anti-oxidative and anti-inflammatory effects are the main mechanisms of BER and CUR. Nuclear factor erythroid-2 related factor 2 (Nrf2) is an important transcription regulator for endogenous antioxidant system. After drug combination interventions, the translocation of Nrf2 from cytoplasm (Fig. 9A) into nucleus (Fig. 9B) was clearly detected and induced downstream protein expression involving quinone oxidoreductase-1 (NQO-1), hemeoxygense-1 (HO-1) and thioredoxin reductase-1 (TrxR-1) (Fig. 9C–E). Obviously, DEAE-DEX@LSDBC exerted the most effective promotion effects on the translocation of Nrf2 to nuclear and the upregulation of NQO-1, HO-1 and TrxR-1 among all the groups, which played a potentially antioxidant role against NAFLD.

On the other hand, BER and CUR combination was verified to inhibit the activation of nuclear factor-kappa B (NF-κB) via suppressing the phosphorylation of p65-a subunit of NF-κB (Fig. 9F) and IκBα-an inhibitor of NF-κB (Fig. 9G). Since phosphorylation of IκBα induces self-degradation that results in phosphorylation of p65 mediated nuclear translocation of NF-κB, the reduced phosphorylation of p65 and IκBα in the treatment groups inhibited the activation of NF-κB. Meanwhile, enhanced oxidative stress in the Model group trigged upregulation of nucleotide-binding oligomerization domain, leucine-rich repeat or pyrin domain-containing 3 (NLRP3), which activated the inflammasome leading to maturation of Caspase-1 and IL-1β (Fig. 9H–J). All the drug combination groups exhibited excellent suppression effects on the levels of NLRP3, Caspase-1 and IL-1β, especially in DEAE-DEX@LSDBC group. Thus, DEAE-DEX@LSDBC exerted anti-inflammatory activity on NAFLD in mice via NF-κB pathway. Moreover, the level of Bax and Bcl-2 were measured to evaluate the apoptosis resulted from oxidative stress and inflammation (Fig. 9K, L). Conspicuously downregulation of Bax and upregulation of Bcl-2 in the treatment groups were also observed in the DEAE-DEX@LSDBC group, which were in constant with the trends in the anti-oxidative and anti-inflammatory effects related pathways.

**Fig. 10:**
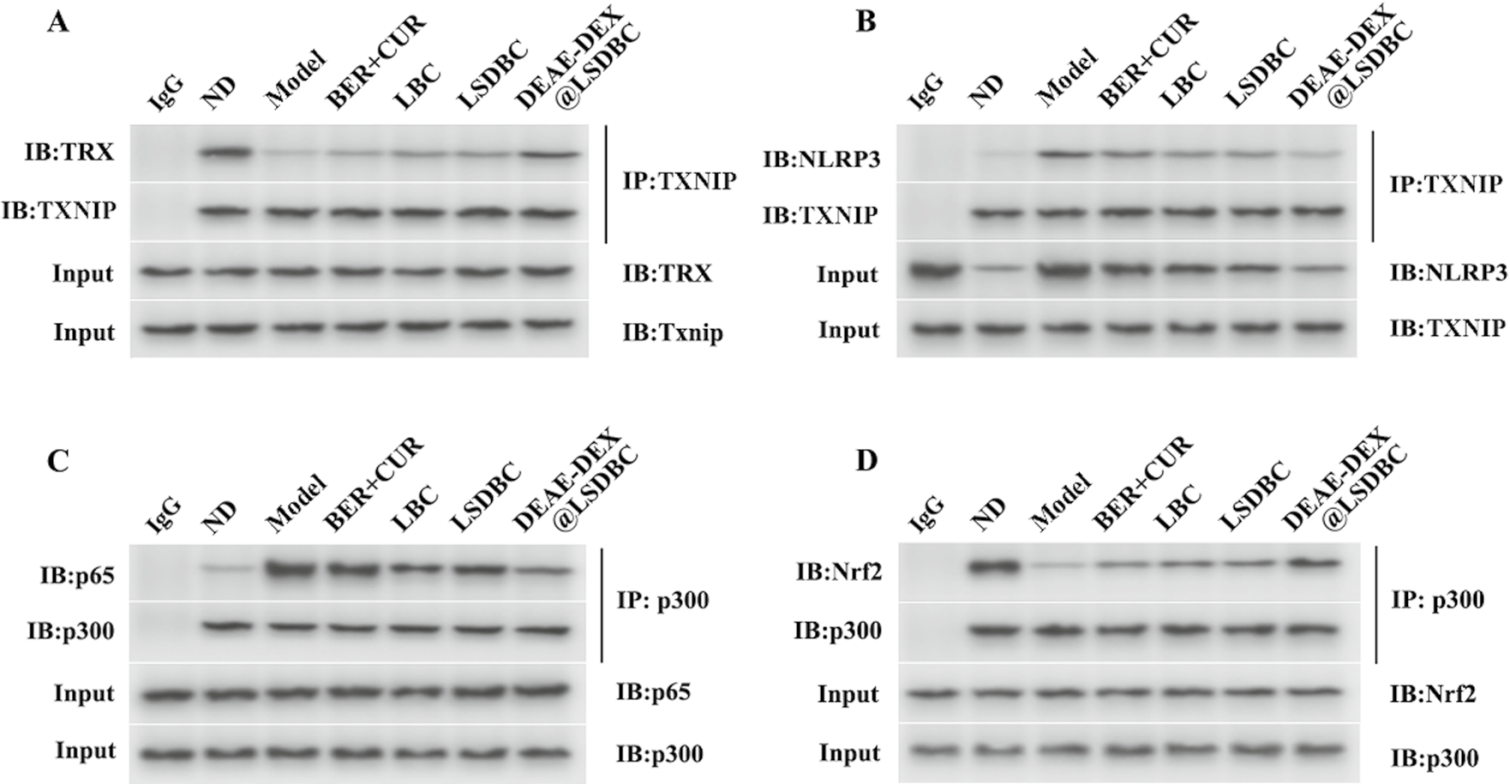
DEAE-DEX@LSDBC promoted TRX-TXNIP and Nrf2–p300 binding. Liver tissue lysates were analyzed for the interaction of TXNIP and **A** TRX or **B** NLRP3 and the interaction of p300 and **C** p65 or **D** Nrf2 by immunoprecipitation

Further analysis of the protein interactions under high fat & sucrose diet and drug combination intervention was studied via coimmunoprecipitation assay. We carried out immunoprecipitation of thioredoxin-interacting protein (TXNIP) and the blot was revealed with TXNIP, thioredoxin (TRX) and NLRP3 antibodies (Fig. 10A, B). After the drug combination treatment, the formation of TXNIP-TRX complex was promoted and TXNIP-NLRP3 inflammasome was inhibited. Similarly, acetyltransferase p300 was immunoprecipitated and the blot was revealed with p300, p65 and Nrf2 antibodies (Fig. 10C, D). The drug combination promoted Nrf2–p300 binding at the expense of p65–p300 interactions, thereby regulating the acetylation of p65 and Nrf2, leading to opposite regulation on the transcriptional activity of p65 and Nrf2. In accordance with the results of protein levels, DEAE-DEX@LSDBC showed the most obvious effects on promoting TXNIP-TRX and p300-Nrf2 formation.

Overall, the results suggested that the BER and CUR combination exerted promising ameliorative effects on NAFLD in mice via anti-oxidative and anti-inflammatory activities. It was closely related with the activation of Nrf2 induced antioxidation effects and suppression of NF-κB induced inflammatory responses. The superior oral absorption, prolonged circulation or synchronized biodistribution of BER and CUR endowed by DEAE-DEX@LSDBC lead to ideal ameliorative effects on NAFLD in mice via enhanced anti-oxidative and anti-inflammatory activities.

## Conclusions

A novel DEAE-DEX coated bilosome with BER and CUR combination encapsulated was developed to ameliorate NAFLD in mice. Compared with free BER  +  CUR, DEAE-DEX@LSDBC improved the stability in the GIT, promoted permeation across mucus and Caco-2 monolayer, enhanced oral absorption and prolonged circulation time of the drug combination. Intriguingly, DEAE-DEX@LSDBC achieved synchronized biodistribution of BER and CUR, especially at liver, which was closely related to the optimal ameliorative effects on NAFLD that mainly mediated by anti-oxidation and anti-inflammation related pathways. Overall, DEAE-DEX@LSDBC was proved to be an effective preparation for the treatment of NAFLD in mice. The DEAE-DEX coated bilosome provided a promising multilayer nanovesicle for oral administration of free drug combinations.

## Methods

### Materials and reagents

Berberine (BER, purity  ≥  98%) and curcumin (CUR, purity  ≥  98%) were obtained from Aladdin Reagent Co., Ltd., (Shanghai, China). Soybean lecithin (SPC) was purchased from Lipoid Co., Ltd., (Ludwigshafen am Rhein, Germany). Cholesterol (CHOL) and octadecylamine (ODA) were provided by Sigma-Aldrich Co., Ltd., (St. Louis, Missouri, USA). Sodium deoxycholate (SDC) and diethylaminoethyl dextran (DEAE-DEX) were obtained from Macklin Biochemical Technology Co., Ltd., (Shanghai, China). Detection kits for total cholesterol (TC, A111), triglyceride (TG, A110), high-density lipoprotein cholesterol (HDL-c, A112), low-density lipoprotein cholesterol (LDL-c, A113), aspartate aminotransferase (AST, C001), alanine aminotransferase (ALT, C009), alkaline phosphatase (ALP, A059), γ-glutamyl transferase (GGT, C017), lactic dehydrogenase (LDH), and glutathione (GSH, A006) were provided by Nanjing Jiancheng Bio-engineering Co., Ltd., (Nanjing, China). Detection kits for malondialdehyde (MDA, S013) and superoxide dismutase (SOD, S0103) were obtained from Beyotime Institute of Biotechnology (Shanghai, China). Detection kits for Oil Red O staining solution, formaldehyde, alcohol, dimethylbenzene, hematoxylin and eosin (H&E) stains were provided by Shenggong Bio-engineering Co., Ltd., (Shanghai, China). The Anti-Nrf2 antibody (12,721), Anti-GAPDH antibody (5174), Anti-histone H3 (4499), Anti-HO-1 antibody (86,806), Anti-phos-p65(Ser536) antibody (3033), Anti-p65 antibody (8242), Anti-phos-IκBα (Ser32) antibody (2859), Anti-IκBα antibody (4812), Anti-IL-1β antibody (12,242), Anti-TXNIP antibody (14,715), Anti-p300 antibody (70,088) were provided by Cell Signaling Technology (Boston, USA). The Anti-NQO-1 antibody (ab28947), Anti-TrxR-1 antibody (ab124954), Anti-NLRP3 antibody (ab263899), Anti-Bax antibody (ab32503), Anti-Bcl-2 antibody (ab182858), Goat Anti-Rabbit IgG H&L (HRP, ab205718), Goat Anti-Mouse IgG H&L (HRP, ab205719), Goat Anti-Rabbit IgG H&L (HRP, ab97051), Anti-Caspase-1 antibody (sc-398715) were provided by Santa Cruz Biotechnology Co., Ltd., (Texas, USA). The inorganic chemicals were obtained from J & K Chemical Co., Ltd., (Beijing, China). Organic solvents of analytical grade were purchased from Sigma-Aldrich Co., Ltd., (St. Louis, Missouri, USA).

### Cells and animals

Caco-2 human colon cancer cell line and LO2 human normal liver cell line were provided by the Cell Bank of the Chinese Academic of Sciences (Shanghai, China). Caco-2 cells and LO2 cells were routinely cultured in DMEM medium (Gibco, California, USA) with 10% (v/v) fetal bovine serum (Gibco, Vienna, Austria), 100 U/mL penicillin and 100 mg/mL streptomycin. The cells were maintained at 37 °C in a humidified atmosphere of 95% air and 5% CO_2_.

Male C57BL/6 J mice (20–25 g) and male Kunming mice (20–25 g) were supplied by Guizhou Medical University Laboratory Animal Co., Ltd., [Guiyang, China, Certificate No. SYXK-(Qian) 2018–0001]. The animals were allowed with free access to water and food for 1-week acclimation. The room temperature was maintained at 23  ±  2 °C with 60–70% humidity with 12-h light/dark cycle.

### Preparation of BER and CUR co-encapsulated bilosomes

The SPC, CHOL, ODA, SDC and CUR in the mass ratio of 20:2:1:3:1 were dissolved in a mixed solvent of chloroform/methanol (4/1, v/v) in around-bottom flask. The solvents were removed dried to form a thin film by a rotary evaporator. Another 2 h under high vacuum was required to remove the remaining solvent. Because BER was encapsulated by a remote loading method, the 0.3 M (NH_4_)_2_SO_4_ solution was added for hydration at 60 °C for 45 min. After sonication in an ice-water bath at 360 W for 5 min (3 s pulse and 3 s pause) with an ultrasonic cell disruptor (650E, Beidi Institute of Scientific Instruments Co., Ltd., Shanghai, China), the CUR-loaded bilosomes were centrifuged at 1500×*g* for 15 min to remove the free CUR. For the encapsulation of BER, the CUR-loaded bilosomes were dialysis (molecular weight cut-off, 12 kDa) against deionized water for 4 h. BER dissolved in deionized water (7 mg/mL) was mixed with the received CUR-loaded bilosomes at BER/SPC of 7/40 (mg/mg). The pH of solution was adjusted by Na_2_CO_3_ (0.1 M) to 7.0 and incubated at 60 °C for 30 min. Unloaded BER was separated from the bilosomes with both BER and CUR encapsulated (LSDBC) through dialysis against PBS (0.01 M, pH 7.4) for 4 h. In the optimization of the formulation, the pH of the external phase in the incubation of BER and the CUR-loaded bilosomes, the incubation time, the mass ratio of SPC/CHOL and SPC/BER were studied in terms of the loading ratio (LR) and loading capability (LC) of both BER and CUR. Besides, the BER and CUR encapsulated liposomes (without SDC inserted) were also prepared as the above optimized procedure and denoted as LBC. After lyophilization, the obtained LBC and LSDBC were stored at 4 °C until use.

### Preparation of DEAE-DEX coated bilosomes

To attach the positively charged DEAE-DEX on the surface of the LBC bilosomes, the same volume of DEAE-DEX in PBS was added dropwise into the bilosomes to different final concentrations (0.13–0.52 mg/mL) in an ice bath, followed by 100 rpm magnetic stirring for 1 h at 25 °C. After filtrating with 0.45 μm membrane, the BER and CUR encapsulated bilosomes with DEAE-DEX coated (DEAE-DEX@LSDBC) was obtained. The concentration of DEAE-DEX was optimized by evaluating the ability of DEAE-DEX@LSDBC to across the mucus layer. The method was described in the “2.7 Permeation across mucus layer in vitro” item. Lastly, the obtained DEAE-DEX@LSDBC were lyophilized and stored at 4 °C until use.

### Characterization of the drug loaded bilosomes

The LR and LC of BER and CUR were determined by a high-performance liquid chromatography (HPLC) with a UV detector (LC-16, Shimadzu Instruments Co., Ltd., Japan). Briefly, 200 μL of the drug loaded bilosomes were added to 9.8 mL of methanol solution and sonicated for 15 min. After filtering through 0.45 μm membrane, the content of CUR loaded was analyzed using a mobile phase of 5% acetic acid/acetonitrile (40:60, v/v) at λ425 nm. For the detection of BER, a mobile phase of 0.1% phosphoric acid/acetonitrile (65:35, v/v) was used and detected at λ345 nm. The mobile phase was delivered at 1 mL/min through a Hanbon C18 column (250  ×  4.6 mm, internal diameter 5 μm). LR and LC were calculated in accordance with the following equations:

1 $${\text{LR }}\left( {{\text{wt}}{\text{. \% }}} \right) = \frac{{\text{weight of loaded drug}}}{{\text{weight of feeding drug}}}\; \times \;100$$

2 $${\text{LC}}\left( {{\text{wt}}{\text{. \% }}} \right) = \frac{{\text{weight of loaded drug}}}{{\text{theoretical total weight of bilosome}}}\; \times \;100$$

The particle size, polydispersity index (PDI) and zeta potential of the prepared preparations were measured by a dynamic laser scattering (90Plus PALS, Brookhaven Instruments Co., Ltd., USA). The Z-average diameter of liposomal preparations was calculated by the automatic mode and the zeta potential was measured more than 10 times run for measurement. The morphology study of LSDBC and DEAE-DEX@LSDBC was implemented with the negative staining method. Film-coated copper grids were used to absorb bilosomes and stained with 1% phosphotungstic acid solution (pH 7.0). The morphology was observed using a transmission electron microscope (TEM, H-7800, Hitachi Co., Ltd., Japan) at an acceleration voltage of 100 kV.

### In vitro drug release behavior

Simulated gastric fluid (SGF) and solutions of intestinal fluid (SIF) were prepared as follows. SGF was prepared by dissolving 16.4 mL of diluted hydrochloric acid and 10 g pepsase in 800 mL of distilled water and then diluted to 1000 mL. For SIF preparation, 6.8 g monobasic potassium phosphate was firstly dissolved in 500 mL distilled water and adjusted to pH 6.8 with 0.1 mol/L NaOH. Then, SIF was prepared by adding 1 g trypsin for every 100 mL of the above solution. Both medium included 0.1% Tween 80 for solubilization of CUR.

Drug release profile of LBC, LSDBC and DEAE-DEX@LSDBC was studied with a membrane dialysis technique in SFG at the first 2 h and then transferred to SIF. Briefly, 1 mL of redissolved preparations were placed in a dialysis bag (molecular weight cut-off, 12 kDa) and dispersed in 20 mL of release medium. In a shaking incubator (100 rpm, 37 °C), the medium (0.1 mL) was collected at predetermined time points for 24 h and the same amount of fresh medium was supplemented. The amount of BER and CUR released in the medium were analyzed by HPLC. All drug release studies meet the requirement of sink condition.

### Permeation across mucus layer in vitro

The permeability of preparations to across the mucus layer were evaluated by the transwell system. Briefly, 0.2 mL of mucin dissolved in hank’s balanced salt solution (HBSS) at 10 mg/mL was added to the upper chambers of transwell (12 insert/24 well plates, pore size 3 μm, Millipore, Billerica, MA, USA) and the lower chambers were filled with 0.8 mL HBSS. Then, 0.2 mL of BER  +  CUR and redissolved LBC, LSDBC and DEAE-DEX@LSDBC (at an equivalent concentration of 90 μg/mL BER and 30 μg/mL CUR) were added into the upper chambers, respectively. Incubating in a water bath at 37 °C and preventing from light, 0.6 mL solution was collected from the lower chambers for measurements at 0.5, 1 and 2 h, respectively. The concentrations of BER and CUR in the lower chambers were determined by HPLC and the ratio of trans-mucus BER and CUR in different groups were also calculated.

### Transcellular permeation assessment on Caco-2 cell monolayers

Caco-2 cells were seeded at a density of 5  ×  10^4^ cells/well in 24-well transwell plates and cultured in DMEM medium with 10% v/v fetal bovine serum for 21 days. The transepithelial electrical resistance (TEER) in Caco-2 cell monolayers was measured by a voltohmmeter (Millicell ERS-2, Millipore Co., Ltd., USA). The TEER values reached to 500 Ω cm^2^ were used in the following experiment. To prepare samples, BER  +  CUR, LBC, LSDBC or DEAE-DEX@LSDBC were respectively dissolved in HBSS with 90 μg/mL BER and 30 μg/mL CUR. Then, the DMEM medium were removed and equilibrated with pre-warmed HBSS for 30 min at 37 °C. The HBSS was replaced with 0.2 mL samples in the upper chambers and 0.8 mL fresh HBSS in the lower chambers. After 2 h incubation, 0.6 mL solution was withdrawn from the lower chambers and quantitatively analyzed by the HPLC for the content of BER and CUR. The apparent permeability (P_app_) of BER and CUR were calculated as the following equation:

3 $$P_{app} = \frac{Q}{{AC_{0} t}}$$

where *Q* was the cumulative amount (ng) of the BER or CUR transported into the lower chambers, *A* was the Caco-2 cell monolayer area (cm^2^), *C*_*0*_ was the BER or CUR concentration (ng/mL) initially in the upper chambers, and *t* was the duration time (s) of the permeability experiment.

Besides, different inhibitors were added in the process of permeability experiments to investigate the transcellular mechanism of DEAE-DEX@LSDBC. Briefly, the culture medium was removed and incubated with pre-warmed HBSS for 30 min. Then, the Caco-2 monolayers were washed and incubated with 4 °C HBSS or 37 °C HBSS containing (1) colchicine (4 μg/mL), (2) chlorpromazine (10 μg/mL), (3) genistein (100 μg/mL), (4) cholic acid (100 μM) and (5) DEAE-DEX (0.39 mg/mL) for 30 min. After that, 0.2 mL of HBSS containing DEAE-DEX@LSDBC were added in the upper chambers at a final concentration of 90 μg/mL BER and 30 μg/mL CUR and the cell monolayers were incubated at 37 °C for another 2 h. Then, the concentration of the BER or CUR transported into the lower chambers was measured with a HPLC. The values of P_app_ were calculated as described above.

### Evaluation on the integrity of Caco-2 cell monolayer

The impact of preparations on the integrity of the Caco-2 monolayers was measured by the TEER values. After equilibrating in HBSS for 30 min, Caco-2 cell monolayers were measured by an electrical resistance system to determine the TEER values. After that, the HBSS was removed and replaced with 0.2 mL HBSS containing BER + CUR, LBC, LSDBC or DEAE-DEX@LSDBC at the concentration of 90 μg/mL BER and 30 μg/mL CUR in the upper chambers and 0.6 mL fresh HBSS in the lower chambers. The TEER values of Caco-2 monolayers in different groups were measured after 0, 1 and 2 h during the incubation. Subsequently, the upper chambers and lower chambers were washed twice with pre-warmed HBSS. The monolayers were incubated in fresh HBSS again and the final TEER values at 4 h were measured.

### Pharmacokinetics assay

The male Kunming mice were randomly divided into four groups, and treated with BER  +  CUR, LBC, LSDBC and DEAE-DEX@LSDBC at dose of 45 mg/kg BER and 15 mg/kg CUR via the oral gavage. At specified time points (0.25, 0.5, 1, 2, 4, 6, 8, 12, 24 h), mice in each group were anesthetized using inhaled isoflurane with a small animal anesthetic equipment (ZS-MV-IV, Zhongshi Scientific Instruments Co., Ltd., Beijing, China). Then the blood was sampled from the postcava and collected in heparin coated tubes. After centrifugation at 1500×*g* for 15 min, 200 μL plasma from the upper layer was mixed with 800 μL methanol and then vortexed 1 min. The mixture was centrifuged at 5000×*g* for 10 min at 4 °C and the supernatant was analyzed using HPLC. The pharmacokinetic data analysis was performed using the pharmacokinetic software WinNonlin 5.2.1 (Pharsight, Mountain View, CA, USA).

### In vivo biodistribution of the preparations

The study was carried out on the male Kunming mice. For the Kunming mice, BER  +  CUR, LBC, LSDBC and DEAE-DEX@LSDBC were administered intragastrically at dose of 45 mg/kg BER and 15 mg/kg CUR. At specified time points (1, 6, 24 h), the mice were anesthetized using small animal anesthetic equipment. The blood was centrifuged to recover the plasma and then analyzed by HPLC. The heart, liver, spleen, lung and kidney were harvested and weighed. After that, organs were homogenized with PBS in a proportion of 3 mL PBS per gram of the liver and 6 mL PBS per gram of the other organs. After centrifugation at 2500×*g* for 10 min, 100 μL supernate of homogenate was mixed with 200 μL methanol and vortexed for 1 min. The final solutions were centrifugated at 10,000×*g* for 10 min and the supernatant was analyzed using HPLC.

### Animal grouping and treatment

After acclimation for 1 week, the male C57BL/6 J mice were divided into six groups: one normal diet group (ND group) and five high fat & sucrose diet groups. The high fat and sucrose diet was made by 40% fat, 40% carbohydrate and 20% protein (D12327, Research Diets, NJ). The ND group was daily treated with saline via the oral gavage for 8 weeks. The five high fat and sucrose diet groups were intragastrically treated with saline (Model group), BER  +  CUR, LBC, LSDBC and DEAE-DEX@LSDBC for 8 weeks continually. All the formulation groups were treated at dose of 45 mg/kg BER and 15 mg/kg CUR. Body weights of mice were recorded every week in the whole course of study.

### Preparation of the tissues and serum samples

After 12 h fasting, the male C57BL/6 J mice were anaesthetized using small animal anesthetic equipment. The blood was freshly collected from the postcava. Subsequently, the serum was separated by centrifugation at 1000×*g* for 10 min and then stored in a container at 4 °C for biochemical analysis. To calculate the body fat rate (BFR), the tissue fats and livers of all groups were excised and weighted. One portion of livers were fixed in 10% phosphate-buffered formalin and the other livers were stored in container at − 80 °C for further analysis.

### Histological assessment

After immersed in 10% formalin solution, the harvested livers were embedded in paraffin blocks and sliced for the H&E staining. The liver sections were stained with Oil-Red-O and counterstained with hematoxylin to observe the hepatic lipid accumulation.

### Biochemical assays

The fasting serum glucose (FSG) level is the glucose metabolism parameter. Serum lipids indicators includes TC, TG, HDL-c, LDL-c. Liver function parameters includes AST, ALT, ALP, GGT and LDH. Antioxidant indicators includes MDA, SOD and GSH. All the biochemical assays were performed according to the kit supplier’s instructions.

### Western blot

Total protein extracts of liver were obtained by homogenizing and centrifuged at 18,000×*g* for 20 min in 4 °C. The loading amount was calculated depended on the protein concentration which was quantified by a BCA protein assay kit (Beyotime Institute of Biotechnology, Jiangsu, China). Protein lysates were separated by a 10% sodium dodecyl sulfate–polyacrylamide gel electrophoresis (SDS-PAGE) and transferred to polyvinylidene fluoride (PVDF) membranes (Millipore Co., Ltd., Bedford, MA, USA). Membranes were blocked by 5% bovine serum albumin (Solarbio, Beijing, China) and incubated overnight with primary antibodies at 4 °C. After that, membranes were washed and incubated with the secondary antibodies. After visualizing with an enhanced chemiluminescence (ECL) kit (7 Sea Biotech, Shanghai, China), digital images of blots were obtained by a GE ImageQuant LAS 500 system (GE Healthcare, Little Chalfont, Buckinghamshire, UK) and analyzed with Image J Software.

### Coimmunoprecipitation assay

Liver tissues were homogenized with a handheld homogenizer with lysis buffer (Beyotime Institute of Biotechnology, P0013) on ice for 30 min and centrifuged at 12,000×*g* at 4 °C for 15 min. The supernatant fractions were collected and incubated with appropriate antibody (1 μg/100 μL) at 4 °C overnight and precipitated with protein A/G-agarose beads (Santa Cruz Biotechnology Co., Ltd., sc-2003) for another 4 h at 4 °C. The beads were washed with the lysis buffer 4 times by centrifugation at 1000×*g* at 4 °C. The immunoprecipitated proteins were separated by SDS–PAGE and western blot was performed with the indicated antibodies.

### Statistical analysis

Results were presented in the form of mean  ±  standard deviation (SD). For statistical comparison among groups, one-way ANOVA analysis with post hoc Tukey–Kramer test was used with GraphPad Prism software package. The *p* value less than 0.05 was statistically significant.

## Supplementary Information

**Additional file 1: Figure S1.** Optimization on the LR of LSDBC in terms of (A) external phase pH and (B) incubation time. The LC of LSDBC was optimized in terms of (C) external phase pH and (D) incubation time. Each bar represents the mean ± SD (*n* = 3). **Figure S2.** Optimization on the LR of LSDBC in terms of (A) the weight ratio of SPC to CHOL and (B) SPC to BER. The LC of LSDBC was optimized in terms of (A) the weight ratio of SPC to CHOL and (B) SPC to BER. Each bar represents the mean ± SD (*n* = 3). **Figure S3. **Transportation of (A) BER and (B) CUR across mucus layer via DEAE-DEX@LSDBC with various amount of DEAE-DEX. Each bar represents the mean ± SD (*n* = 3). **Figure S4.** UV spectrum of BER, CUR, LIP, LBC, LSDBC and DEAE-DEX@LSDBC. **Figure S5. **Cytotoxicity of preparations against Caco-2 cells and LO2 cells. Each bar represents the mean ± SD (*n* = 3). **Figure S6.** Synchronized biodistribution of BER and CUR from preparations orally delivery in the male Kunming mice. The T/P ratios for BER and CUR from (A–C) BER+CUR, (D–F) LBC, (G–I) LSDBC and (J–L) DEAE-DEX@LSDBC in the heart, liver, spleen, lung and kidney at (A, D, G, J) 1 h, (B, E, H, K) 6 h and (C, F, I, L) 24 h after orally administration. Each bar represents the mean ± SD (*n* = 3). ^*^p < 0.05, ^**^p < 0.01 and ^***^p < 0.001 BER *vs* CUR in various tissues. **Figure S7. **Representative morphological images of Oil-Red-O staining on LO2 cells after 12 h incubation with various preparations (Scar bar 50 μm). **Figure S8. **Drug release behavior of CUR and BER in both SIF and SGF medium for 24 h. Each symbol represents the mean ± SD (n = 3). **Table S1. **Pharmacokinetics parameters of BER after oral administration (*n* = 5). **Table S2. **Pharmacokinetics parameters of CUR after oral administration (*n* = 5).

## Data Availability

All data generated or analyzed during this study are included in this published article and its supplementary information file.

## Abbreviations

ACWC: Animal Care Welfare Committee
ALP: Alkaline phosphatase
ALT: Alanine aminotransferase
AMPK: Adenosine monophosphate activated protein kinase
ASGPR: Asialoglycoprotein receptor
AST: Aspartate aminotransferase
BER: Berberine
BFR: Body fat rate
CHOL: Cholesterol
CUR: Curcumin
DEAE-DEX: Diethylaminoethyl dextran
DEAE-DEX@LSDBC: DEAE-DEX-coated bilosome with BER and CUR encapsulated
ECL: Enhanced chemiluminescence
FSG: Fasting serum glucose
GGT: γ-Glutamyl transferase
GIT: Gastrointestinal tract
GSH: Glutathione
HBSS: Hank’s balanced salt solution
HCC: Hepatocellular carcinoma
HDL-c: High-density lipoprotein cholesterol
HE: Hematoxylin and eosin
HO-1: Hemeoxygense-1
HPLC: High-performance liquid chromatography
LBC: The BER and CUR encapsulated liposomes without SDC inserted
LC: Loading capability
LDH: Lactic dehydrogenase
LDL-c: Low-density lipoprotein cholesterol
LDLR: Low density lipoprotein receptor
LR: Loading ratio
LSDBC: Bilosomes with both BER and CUR encapsulated
MDA: Malondialdehyde
NAFLD: Non-alcohol fatty liver disease
NASH: Nonalcoholic steatohepatitis
NF-κB: Nuclear factor-kappa B
NLRP3: Pyrin domain-containing 3
NQO-1: Quinone oxidoreductase-1
Nrf2: Nuclear factor erythroid-2 related factor 2
ODA: Octadecylamine
P_app_: Apparent permeability
PDI: Polydispersity index
PVDF: Polyvinylidene fluoride
SDC: Sodium deoxycholate
SDS-PAGE: Sulfate–polyacrylamide gel electrophoresis
SGF: Simulated gastric fluid
SIF: Solutions of intestinal fluid
SOD: Superoxide dismutase
SPC: Soybean lecithin
TC: Total cholesterol
TEER: Transepithelial electrical resistance
TEM: Transmission electron microscope
TG: Triglyceride
TRX: Thioredoxin
TrxR-1: Thioredoxin reductase-1
TXNIP: Thioredoxin-interacting protein
