# Supplementary material for: Improving the ameliorative effects of berberine and curcumin combination via dextran-coated bilosomes on non-alcohol fatty liver disease in mice
Source: J Nanobiotechnology. 2021 Aug 4;19:230. doi: 10.1186/s12951-021-00979-1 (PMC8336351; doi:10.1186/s12951-021-00979-1)
Supplement: Supplementary file 1 — Additional file 1: Figure S1. Optimization on the LR of LSDBC in terms of (A) external phase pH and (B) incubation time. The LC of LSDBC was optimized in terms of (C) external phase pH and (D) incubation time. Each bar represents the mean ± SD (n = 3). Figure S2. Optimization on the LR of LSDBC in terms of (A) the weight ratio of SPC to CHOL and (B) SPC to BER. The LC of LSDBC was optimized in terms of (A) the weight ratio of SPC to CHOL and (B) SPC to BER. Each bar represents the mean ± SD (n = 3). Figure S3. Transportation of (A) BER and (B) CUR across mucus layer via DEAE-DEX@LSDBC with various amount of DEAE-DEX. Each bar represents the mean ± SD (n = 3). Figure S4. UV spectrum of BER, CUR, LIP, LBC, LSDBC and DEAE-DEX@LSDBC. Figure S5. Cytotoxicity of preparations against Caco-2 cells and LO2 cells. Each bar represents the mean ± SD (n = 3). Figure S6. Synchronized biodistribution of BER and CUR from preparations orally delivery in the male Kunming mice. The T/P ratios for BER and CUR from (A–C) BER+CUR, (D–F) LBC, (G–I) LSDBC and (J–L) DEAE-DEX@LSDBC in the heart, liver, spleen, lung and kidney at (A, D, G, J) 1 h, (B, E, H, K) 6 h and (C, F, I, L) 24 h after orally administration. Each bar represents the mean ± SD (n = 3). *p < 0.05, **p < 0.01 and ***p < 0.001 BER vs CUR in various tissues. Figure S7. Representative morphological images of Oil-Red-O staining on LO2 cells after 12 h incubation with various preparations (Scar bar 50 μm). Figure S8. Drug release behavior of CUR and BER in both SIF and SGF medium for 24 h. Each symbol represents the mean ± SD (n = 3). Table S1. Pharmacokinetics parameters of BER after oral administration (n = 5). Table S2. Pharmacokinetics parameters of CUR after oral administration (n = 5). [file 12951_2021_979_MOESM1_ESM.docx]

**Supplementary Information**

Improving the Ameliorative Effects of Berberine and Curcumin Combination via Dextran-coated Bilosomes on Non-alcohol Fatty Liver Disease in Mice

Yi Chen ^1,2^, Zhaohui Jiang ^5^, Jinzhuan Xu ^2^, Jiyuan Zhang ^4^, Runbin Sun ^6^, Jia Zhou ^2^, Yuan Lu ^1^, Zipeng Gong ^1^, Jing Huang ^1^, Xiangchun Shen ^1,2^, Qianming Du ^3,4,*^, Jianqing Peng ^1,2,*^

^1^ State Key Laboratory of Functions and Applications of Medicinal Plants, Guizhou Medical University, Guiyang 550014, China.

^2^ Key Laboratory of Optimal Utilization of Natural Medicine Resources, School of Pharmaceutical Sciences, Guizhou Medical University, Guiyang 550025, China.

^3^ General Clinical Research Center, Nanjing First Hospital, Nanjing Medical University, Nanjing 210006, China.

^4^ Department of Clinical Pharmacy, School of Basic Medicine & Clinical Pharmacy, China Pharmaceutical University, Nanjing 210009, China.

^5^ Department of Clinical Laboratory, The first People’s Hospital of Guiyang, Guiyang 550002, China.

^6^ Nanjing Drum Tower Hospital, the Affiliated Hospital of Nanjing University Medical School, Nanjing 210008, China.

**Supplementary Methods**

**1. Cytotoxicity on Caco-2 cells and LO2 cells**

Caco-2 cells and LO2 cells were seeded in 96 well plates at a density of 1 × 10^4^ cells/well and incubated at 37 °C with 5% CO_2_ and 95% relative humidity. After 24 h incubation, the growth medium was changed into serum-free medium containing BER, CUR, BER+CUR, LBC, LSDBC and DEAE-DEX@LSDBC at various concentration of BER and CUR as well as blank carrier (DEAE-DEX@Bilosome). After 24 h incubation, the medium was replaced by 100 μL serum-free medium containing 10% (v/v) Cell Counting Kit-8 (CKK-8) and incubated for another 1 h in the dark. The optical density (OD) of the plates was measured on a microplate reader (Infinite m200 pro, TECAN, Männedorf, Switzerland) at 450 nm. The cell inhibition rate (%) was calculated according to the formula (1):

$Inhibition rate \left( \% \right)=(1-\frac{\mathrm{OD}_{\mathrm{sample}}-\mathrm{OD}_{\mathrm{contral}}}{\mathrm{OD}_{\mathrm{normal}}-\mathrm{OD}_{\mathrm{contral}}})\times100$ (1)

**2. Oil-red-O staining on** **free fatty acids (FFA) treated LO2 cells**

LO2 cells were incubated in a 24-well plate at a density of 1 × 10^4^ cells/well at 37 °C with 5% CO_2_ overnight. The culture medium containing a mixture of long-chain FFA (oleate/palmitate, C18:1/C16:0, 2:1) at concentration of 400 μM was added and incubate for 12 h. Untreated cells were used as a control. Then the medium was replaced by PBS, BER, CUR, BER+CUR, LBC, LSDBC and DEAE-DEX@LSDBC at an equivalent concentration of BER 3 μg/ml and CUR 1 μg/ml and incubated for another 12 h. The treated cells were washed with PBS and fixed by 10% formalin for 30 min. Oil-Red-O was added and washed by 75% ethanol after 1 h. Cells were then stained with hematoxylin reagent for 30 s. After washed three times with distilled water, the cells were observed under a microscope.

**Supplementary Figures**


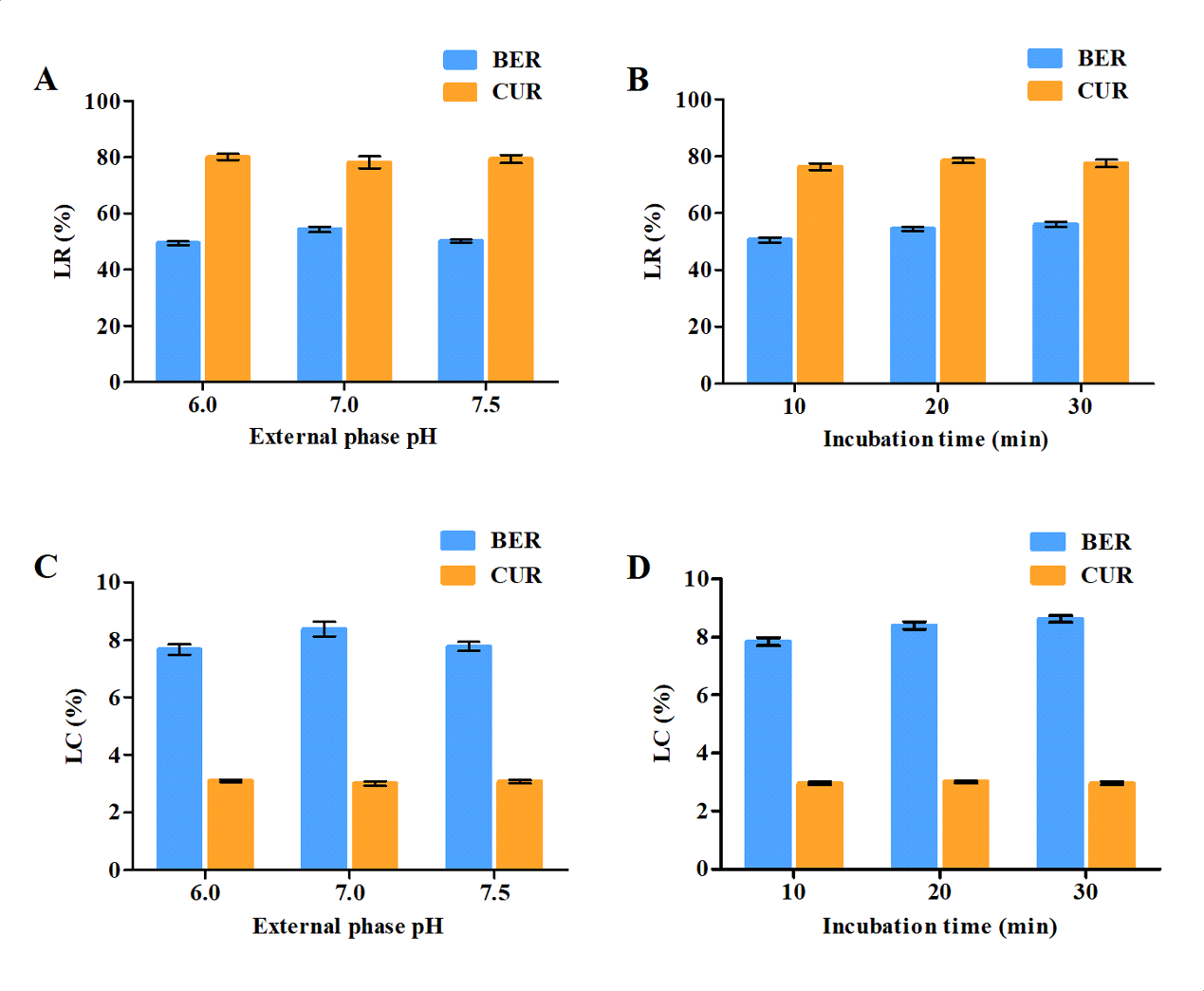


**Figure S1** Optimization on the LR of LSDBC in terms of (A) external phase pH and (B) incubation time. The LC of LSDBC was optimized in terms of (C) external phase pH and (D) incubation time. Each bar represents the mean ± SD (*n* = 3).


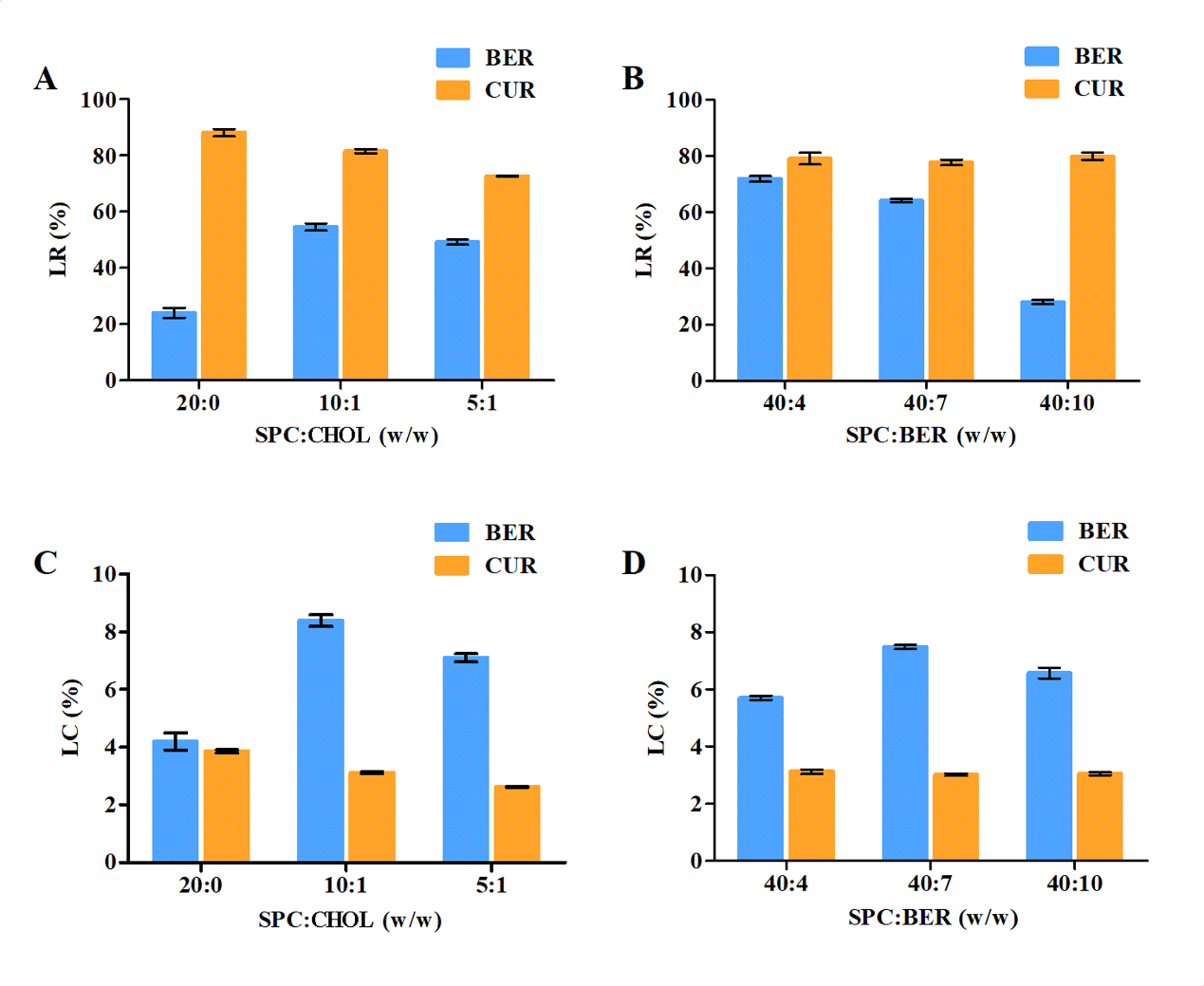


**Figure S2** Optimization on the LR of LSDBC in terms of (A) the weight ratio of SPC to CHOL and (B) SPC to BER. The LC of LSDBC was optimized in terms of (A) the weight ratio of SPC to CHOL and (B) SPC to BER. Each bar represents the mean ± SD (*n* = 3).


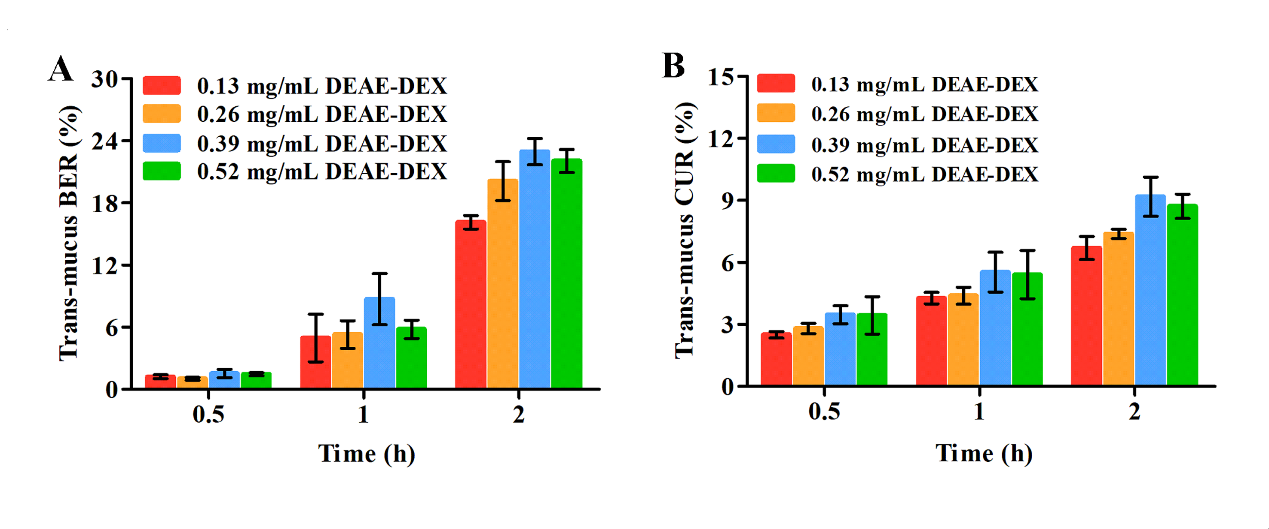


**Figure S3** Transportation of (A) BER and (B) CUR across mucus layer via DEAE-DEX@LSDBC with various amount of DEAE-DEX. Each bar represents the mean ± SD (*n* = 3).





**Figure S4** UV spectrum of BER, CUR, LIP, LBC, LSDBC and DEAE-DEX@LSDBC.


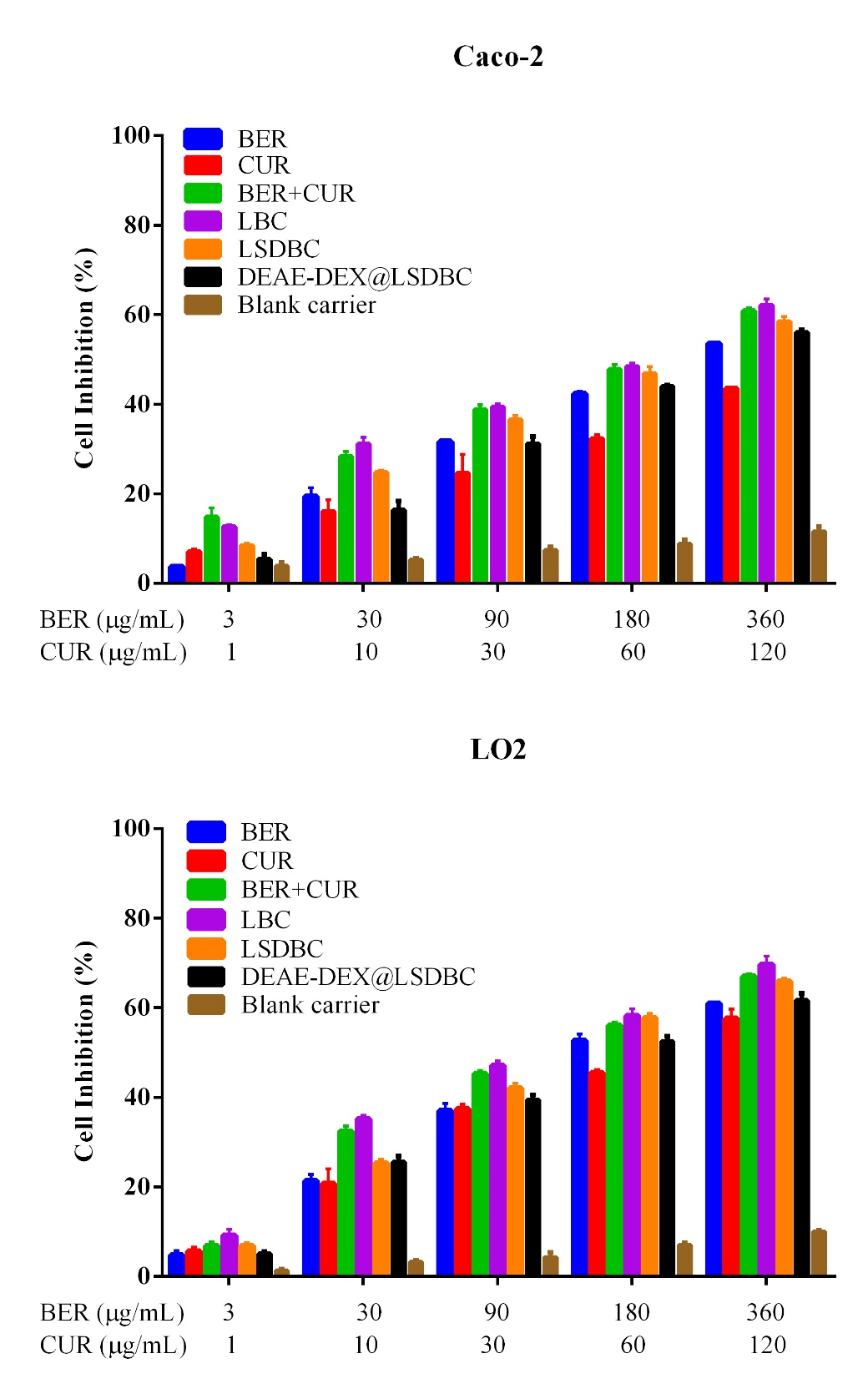


**Figure S5** Cytotoxicity of preparations against Caco-2 cells and LO2 cells. Each bar represents the mean ± SD (*n* = 3).


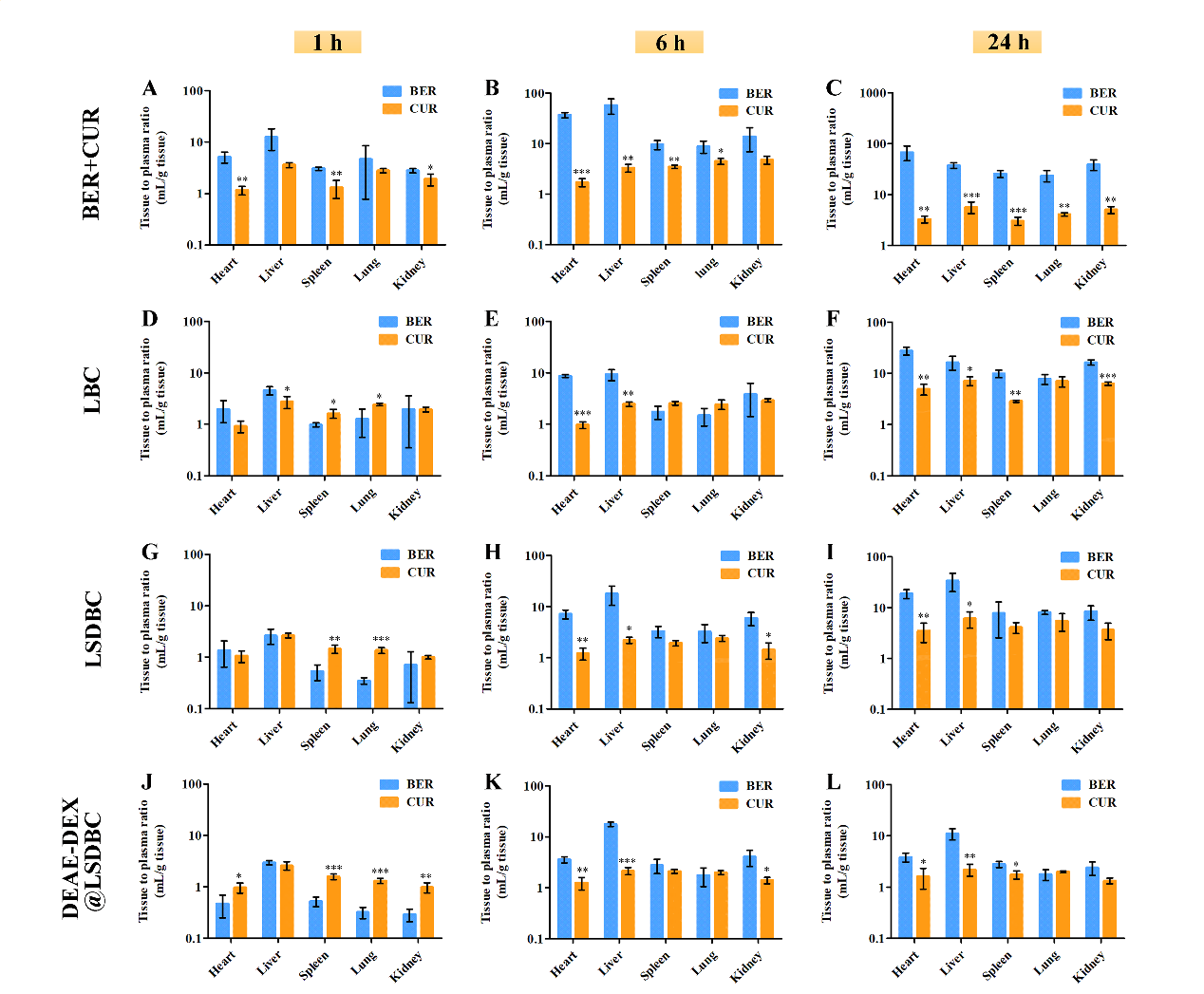


**Figure S6** Synchronized biodistribution of BER and CUR from preparations orally delivery in the male Kunming mice. The T/P ratios for BER and CUR from (A–C) BER+CUR, (D–F) LBC, (G–I) LSDBC and (J–L) DEAE-DEX@LSDBC in the heart, liver, spleen, lung and kidney at (A, D, G, J) 1 h, (B, E, H, K) 6 h and (C, F, I, L) 24 h after orally administration. Each bar represents the mean ± SD (*n* = 3). ^*^*p* < 0.05, ^**^*p* < 0.01 and ^***^*p* < 0.001 BER *vs* CUR in various tissues.


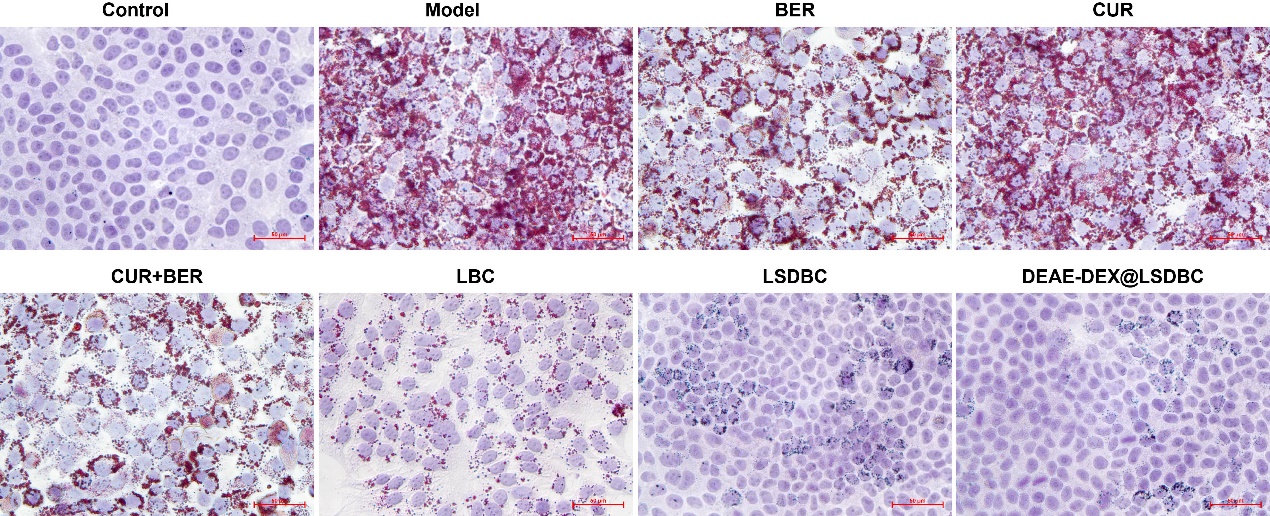


**Figure S7** Representative morphological images of Oil-Red-O staining on LO2 cells after 12 h incubation with various preparations (Scar bar 50 μm).

**
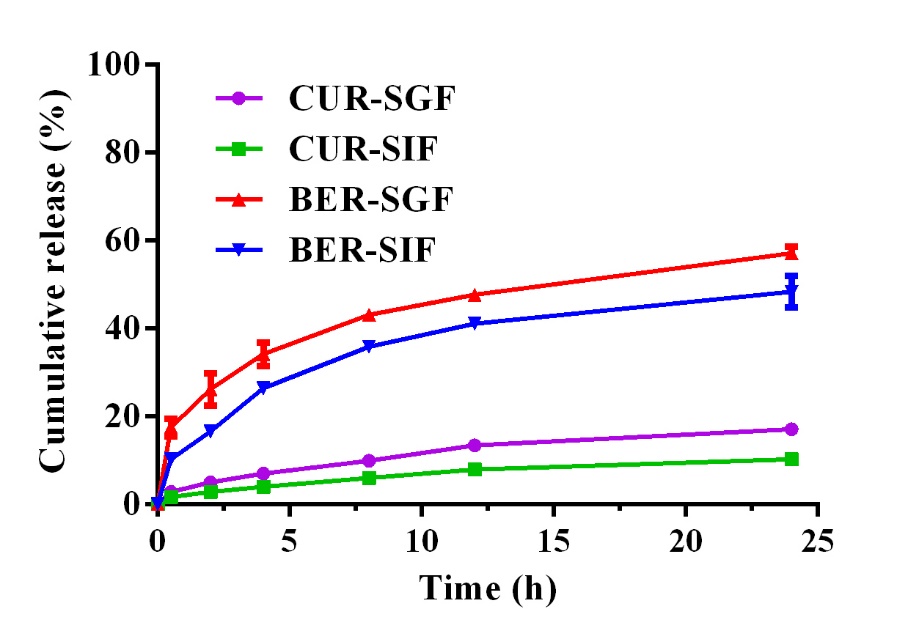
**

**Figure S8** Drug release behavior of CUR and BER in both SIF and SGF medium for 24 h. Each symbol represents the mean ± SD (n = 3).

**Supplementary Tables**

**Table S1** Pharmacokinetics parameters of BER after oral administration (*n* = 5)

| Parameters | BER + CUR | LBC | LSDBC | DEAE-DEX@LSDBC |
| --- | --- | --- | --- | --- |
| T_1/2_ (h) | 1.60 ± 0.33 | 6.80 ± 2.11 | 8.24 ± 2.05 | 15.48 ± 13.81^*^ |
| T_max_ (h) | 0.90 ± 0.22 | 1.30 ± 0.67 | 0.70 ± 0.27 | 0.90 ± 0.22 |
| C_max_ (ng/mL) | 12.78 ± 2.68 | 57.25 ± 11.47 | 320.18 ± 25.76^***, ###^ | 406.76 ± 67.51^***, ###,&&^ |
| AUC_0-t_ (h*ng/mL) | 51.74 ± 4.18 | 385.13 ± 56.63^*^ | 847.35 ± 94.58^***,##^ | 1353.89 ± 306.71^***,###,&&&^ |
| AUC_0-∞_(h*ng/mL) | 52.21 ± 4.03 | 419.58 ± 78.32 | 895.94 ± 94.92^***,#^ | 1750.78 ± 448.30^***,###,&&&^ |
| BA (%) | 100.00 ± 7.72 | 803.69 ± 150.01 | 1716.13 ± 181.81^***,#^ | 3353.5 ± 858.7^***,###,&&&^ |
| Cl/F (L/h/kg) | 865.93 ± 64.67 | 109.96 ± 18.31^***^ | 50.69 ± 5.55^***^ | 27.02 ± 6.47 ^***,##^ |
| MRT (h) | 3.21 ± 0.20 | 8.81 ± 2.38 | 5.54 ± 0.84 | 16.29 ± 13.97^*^ |

*: *p* < 0.05, **: *p* < 0.01 and ***: *p* < 0.001, statistically significant *vs* BER+CUR.

^#^: *p* < 0.05, ^##^: *p* < 0.01 and ^###^: *p* < 0.001, statistically significant *vs* LBC.

^&^: *p* < 0.05, ^&&^: *p* < 0.01 and ^&&&^: *p* < 0.001, statistically significant *vs* LSDBC group.

**Table S2** Pharmacokinetics parameters of CUR after oral administration (*n* = 5)

| Parameters | BER + CUR | LBC | LSDBC | DEAE-DEX@LSDBC |
| --- | --- | --- | --- | --- |
| T_1/2_ (h) | 1.55 ± 0.16 | 6.18 ± 1.34 | 11.68 ± 4.66^*^ | 25.41 ± 18.53^**^ |
| T_max_ (h) | 0.45 ± 0.11 | 0.60 ± 0.22 | 0.60 ± 0.22 | 1.80 ± 0.45^***,###,&&&^ |
| C_max_ (ng/mL) | 61.22 ± 4.59 | 88.39 ± 14.62 | 146.38 ± 41.13^*^ | 167.51 ± 70.65^**,#^ |
| AUC_0-t_ (h*ng/mL) | 265.09 ± 23.02 | 598.17 ± 95.84^*^ | 1177.40 ± 86.44^***, ###^ | 1635.97 ± 217.74^***,###,&&^ |
| AUC_0-∞_(h*ng/mL) | 268.55 ± 23.65 | 647.01 ± 121.02 | 1557.66 ± 302.42^**, #^ | 3416.5 ±1604.12^***,###,&^ |
| BA (%) | 100.00 ± 8.81 | 240.92 ± 45.06 | 580.02 ± 112.61 | 1272.19 ± 597.32^***,###,&^ |
| Cl/F (L/h/kg) | 56.21 ± 5.04 | 23.85 ± 4.45^***^ | 9.92 ± 1.91^***, ###^ | 5.00 ± 1.70^***, ###^ |
| MRT (h) | 3.97 ± 0.15 | 9.26 ± 1.63 | 16.40 ± 6.44 | 36.77 ± 26.81^**^ |

*: *p* < 0.05, **: *p* < 0.01 and ***: *p* < 0.001, statistically significant *vs* BER+CUR.

^#^: *p* < 0.05, ^##^: *p* < 0.01 and ^###^: *p* < 0.001, statistically significant *vs* LBC.

^&^: *p* < 0.05, ^&&^: *p* < 0.01 and ^&&&^: *p* < 0.001, statistically significant *vs* LSDBC group.
